# A multicenter, randomized controlled trial of individualized occupational therapy for patients with schizophrenia in Japan

**DOI:** 10.1371/journal.pone.0193869

**Published:** 2018-04-05

**Authors:** Takeshi Shimada, Manami Ohori, Yusuke Inagaki, Yuko Shimooka, Naoya Sugimura, Ikuyo Ishihara, Tomotaka Yoshida, Masayoshi Kobayashi

**Affiliations:** 1 Department of Occupational Therapy, Medical Corporation Seitaikai, Mental Support Soyokaze Hospital, Nagano, Japan; 2 Department of Health Sciences, Graduate School of Medicine, Shinshu University, Nagano, Japan; 3 Department of Occupational Therapy, North Alps Medical Center, Azumi Hospital, Nagano, Japan; 4 Department of Occupational Therapy, Nagano Prefectural Mental Wellness Center Komagane, Nagano, Japan; 5 Department of Occupational Therapy, Social Medical Corporation Ritsuzankai, Iida Hospital, Nagano, Japan; 6 Department of Occupational Therapy, Medical Corporation Akitsukai, Nanshin Hospital, Nagano, Japan; 7 Department of Occupational Therapy, Medical Corporation Aiseikai, Matsuoka Hospital, Nagano, Japan; TNO, NETHERLANDS

## Abstract

The individualized occupational therapy (IOT) program is a psychosocial program that we developed to facilitate proactive participation in treatment and improve cognitive functioning and other outcomes for inpatients with acute schizophrenia. The program consists of motivational interviewing, self-monitoring, individualized visits, handicraft activities, individualized psychoeducation, and discharge planning. This multicenter, open-labeled, blinded-endpoint, randomized controlled trial evaluated the impact of adding IOT to a group OT (GOT) program as usual for outcomes in recently hospitalized patients with schizophrenia in Japanese psychiatric hospitals setting compared with GOT alone. Patients with schizophrenia were randomly assigned to the GOT+IOT group or the GOT alone group. Among 136 randomized patients, 129 were included in the intent-to-treat population: 66 in the GOT+IOT and 63 in the GOT alone groups. Outcomes were administered at baseline and discharge or 3 months following hospitalization including the Brief Assessment of Cognition in Schizophrenia Japanese version (BACS-J), the Schizophrenia Cognition Rating Scale Japanese version, the Social Functioning Scale Japanese version, the Global Assessment of Functioning scale, the Intrinsic Motivation Inventory Japanese version (IMI-J), the Morisky Medication Adherence Scale-8 (MMAS-8), the Positive and Negative Syndrome Scale (PANSS), and the Japanese version of Client Satisfaction Questionnaire-8 (CSQ-8J). Results of linear mixed effects models indicated that the IOT+GOT showed significant improvements in verbal memory (p <0.01), working memory (p = 0.02), verbal fluency (p < 0.01), attention (p < 0.01), and composite score (p < 0.01) on the BACS-J; interest/enjoyment (p < 0.01), value/usefulness (p < 0.01), perceived choice (p < 0.01), and IMI-J total (p < 0.01) on the IMI-J; MMAS-8 score (p < 0.01) compared with the GOT alone. Patients in the GOT+IOT demonstrated significant improvements on the CSQ-8J compared with the GOT alone (p < 0.01). The present findings provide support for the feasibility in implementing an IOT program and its effectiveness for improving cognitive impairment and other outcomes in patients with schizophrenia.

## Introduction

Psychosocial treatments designed to remediate or to bypass cognitive impairments are important to pursue to maximize outcomes for patients with schizophrenia [1–6]. Cognitive impairment is a core feature of schizophrenia [7–10], and previous studies have demonstrated that it is strongly correlated with daily functioning and other functional outcomes [7–12]. Because of the importance of cognitive impairment in schizophrenia, it has been proposed as an appropriate target for intervention [4, 5, 11]. Antipsychotic treatment reduces symptoms of schizophrenia; however, it does little to improve cognitive impairment [5, 13, 14]. Therefore, it is important to improve cognitive impairment in patients with schizophrenia using psychosocial treatment [5].

In Japan, occupational therapy (OT) is a required component of psychosocial treatment that helps therapists provide short-term, inpatient care for patients with acute schizophrenia. Furthermore, it is believed that individualized OT (IOT) is needed for the achievement of this goal. However, the existing medical fee system for psychiatric OT in Japan assumes that group treatment is the standard. For this reason, an activity-oriented group OT (GOT) intervention is widely practiced for patients with schizophrenia in several Japanese psychiatric hospitals, and individualized OT is not implemented often in Japan [15].

Several studies have examined the effects of OT on patients with schizophrenia. Many of these reported that OT can improve symptoms, and combined interventions of OT and pharmacological treatment can more effectively improve symptoms than pharmacological treatment alone [16, 17]. However, the effectiveness of OT for cognitive impairment in patients with schizophrenia has not been sufficiently verified.

The IOT program is an intervention that we developed as the original OT program based on individualized interventions for inpatients with acute schizophrenia, which facilitates proactive participation in treatment and improving outcomes [18]. There are no similar IOT program as far as we know, and the its evidence is not sufficient. IOT strategies are tailored to enhance cognitive functioning and prompt adaptive behaviors to maximize the functional outcomes of patients with schizophrenia. It consists of a combination of effective psychosocial treatment programs that are very relevant to OT practice, including motivational interviewing, self-monitoring, individualized visits, handicraft activities, individualized psychoeducation, and discharge planning. It was implemented on a one-on-one basis with occupational therapists. The component of the program specific to OT profession was to incorporate craft activities with the intention of improving cognitive impairment in schizophrenia. In the program, we used constitutive handicraft activities with the individualized coaching of occupational therapists as a means of improving the cognitive impairment associated with schizophrenia. Handicraft activities have been widely used as OT therapeutic modalities [19–21].

Our finding of a previous pilot study concerning the IOT program provided preliminary support for the feasibility of IOT for helping patients with schizophrenia who were enrolled in an OT program at a Japanese psychiatric hospital setting and its beneficial effects on improving cognitive impairment and symptoms for patients with schizophrenia [18]. However, it had several limitations, including being conducted in only one site and having selection biases because the study design was a non-randomized controlled trial where participants were assigned to either the IOT + GOT or GOT alone group by voluntary selection according to their preferences. In addition, the outcomes used in the study were not sufficient to examine the effects of IOT because only cognitive functioning, social functioning, and symptoms were measured.

Therefore, the present study reexamined the feasibility and effectiveness of IOT for cognitive functioning and other outcomes including social functioning, intrinsic motivation, medication adherence, symptoms, and treatment satisfaction in patients with schizophrenia using a multicenter, randomized, controlled trial design. We hypothesized that IOT would be feasible for use in Japanese psychiatric hospitals settings and improve cognitive impairment as compared with GOT. Moreover, hypotheses included that other outcomes would be better for patients in IOT in comparison to those in GOT.

## Methods

This study was a multicenter, open-labeled, blinded-endpoint, randomized controlled trial evaluating the impact of adding IOT to GOT for patients with schizophrenia. The study took place between February 2016 and March 2017 during an OT program affiliated with Japanese psychiatric hospitals, including one public hospital and five private hospitals in Nagano Prefecture, Japan, which is a suburban community. Treatment in this study lasted approximately 3 months from hospitalization to discharge. All study procedures were approved by the ethics committee of Shinshu University (No 3256), Medical Corporation Seitaikai Mental Support Soyokaze Hospital, North Alps Medical Center Azumi Hospital, Nagano Prefectural Mental Wellness Center Komagane, Social Medical Corporation Ritsuzankai Iida Hospital, Medical Corporation Aiseikai Matsuoka Hospital, and Medical Corporation Akitsukai Nanshin Hospital. Study participants provided written, informed consent for all study procedures. The study was registered in the University Hospital Medical Information Network Clinical Trials Registry (UMIN-CTR) (UMIN000019569).

### Participants

Participants were recruited through referrals from occupational therapists at each study center. Inclusion criteria were aged 20–65 years; recently hospitalized patients in a psychiatric hospital; with a diagnosis of schizophrenia or schizoaffective disorder based on a structured clinical interview using the Diagnostic and Statistical Manual of Mental Disorders, 5th edition’s criteria [22]; and eligibility of the study was assessed by the occupational therapist and psychiatrist in charge. Exclusion criteria were a diagnosis of mental retardation or alcohol or drug disorders (abuse or dependence); any current or history of neurological disorders including head injury, cerebral vascular disorders, epilepsy, or dementia; and the need for a specific individual intervention for a physical dysfunction.

### Measures

Assessments were conducted by trained evaluators who were blind to treatment assignment before assignment (baseline assessment) and at discharge or 3 months following hospitalization (post assessment).

Demographics including age, sex, diagnosis, age of onset, number of hospital stays, total length of hospital stays, education, employment experience, marital status, OT experience, length of time from hospitalization to OT intervention implementation, length of OT intervention, number of OT sessions, and antipsychotic (chlorpromazine equivalent) dose were drawn from participants, support persons’ interviews, and medical record review.

Cognitive functioning was assessed with the Brief Assessment of Cognition in Schizophrenia Japanese version (BACS-J) [23, 24] and the Schizophrenia Cognition Rating Scale Japanese version (SCoRS-J) [25–27]. The BACS-J assesses multiple aspects in cognitive functioning of schizophrenia and includes six measures: verbal memory, working memory, motor speed, verbal fluency, attention, and executive functioning. Each of the six measures was standardized by creating z-scores, whereby the mean scores of the healthy participants were set to zero, and the standard deviations were set to one. The composite score was calculated by averaging all the z-scores of the six BACS-J measures. The SCoRS-J is a 20-item interview-based measure of cognitive impairment with questions aimed at the degree that this impairment affects daily functioning [25–27]. A global rating, which reflects the overall impression of patients’ level of cognitive difficulty in 20 areas of cognition, is also generated. The items were developed to assess the cognitive domains of memory, learning, attention, working memory, problem solving, motor skills, social cognition, and language production. Each item is rated on a scale ranging from 1–4 and the global rating is rated 1–10, with higher ratings reflecting more impairment. The anchor points for each item focus on the degree of impairment in that ability and the degree that the deficit impairs daily functioning. Evaluators considered cognitive deficits only and attempted to rule out non-cognitive sources of the deficits. Complete administration of the SCoRS-J included three different ratings: a rating based on an interview with the patient, a rating based on an interview with an informant (i.e., the person who had the most regular contact with the patient in everyday situations), and a rating generated by the interviewer who administered the scale to the patient and informant. A simple sum of the 20 SCoRS-J items was referred to as the SCoRS-J total. In this study, we used an interview with the patient form to assess subjective cognitive deficits and the degree that they affected daily functioning.

Prehospital social functioning was assessed with the Social Functioning Scale Japanese version (SFS-J) [28, 29] at baseline. The SFS-J is a 79-item self-report questionnaire designed to assess social functioning essential to the successful community maintenance of patients with schizophrenia and has 7 sub-scores: withdrawal/social engagement, interpersonal communication, pro-social activities, recreation, independence-competence, independence-performance, and employment/occupation. A higher score indicates a higher level of social functioning.

Social functioning was assessed with the Global Assessment of Functioning (GAF) scale [30]. The GAF scale is a single-item rating scale for the measurement of patients’ psychological, social, and occupational functioning. Score on this scale was rated between 0 and 100, with each 10-point segment being defined in relation to levels of functioning, with higher scores indicating better functioning.

Intrinsic motivation was assessed with the Intrinsic Motivation Inventory Japanese version (IMI-J) [31]. The IMI-J is a 21-item self-report scale that measures interest/enjoyment, value/usefulness, and perceived choice. Items were answered on a 7-point Likert scale with responses ranging from ‘‘*not at all true*” to ‘‘*very true*” and a higher total score reflected greater intrinsic motivation for a specified task.

Medication adherence was assessed with the eight-item Morisky Medication Adherence Scale (MMAS-8) [32, 33]. The MMAS-8 comprises 8 self-report items. Response categories are *yes/no* for each item with a dichotomous response and a 5-point Likert response for the last item resulting in a total score that ranges from 0 to 8, with higher scores indicating better adherence to the prescribed medications.

Symptoms were assessed with the Positive and Negative Syndrome Scale (PANSS) [34]. The PANSS is a 30-item rating scale designed to assess the severity of psychotic symptoms. Outcomes on the PANSS were analyzed using the 3-factor solution, which included positive, negative, and general psychopathology. All items were rated 1 (*absent*) to 7 (*extreme*), with higher scores indicating more severe symptoms.

Treatment satisfaction was assessed with the Japanese version of the Client Satisfaction Questionnaire (CSQ-8J) [35, 36] at discharge or 3 months following hospitalization. The CSQ-8J is an 8-item self-report questionnaire that is answered using a 4-point Likert design (1–4). Total scores range from 8 to 32 and higher scores indicate greater treatment satisfaction.

### Interventions

Time and frequency of OT implementation for both the GOT + IOT and GOT only groups were adjusted according to recovery progress; however, these were generally 1–2 hours at a time, 3–5 times per week. Notably, more than half of the OT time was devoted to IOT among the GOT + IOT group. Occupational therapists provided support, such as consultations concerning living challenges, preparation support for discharge, and provision of information concerning available social resources and community services for all participants, regardless of the type of OT program (GOT + IOT versus GOT alone). In addition, support personnel, including hospital staff other than the occupational therapists, provided necessary support for all the participants, regardless of the type of OT program.

#### IOT program

The devised IOT program aimed to facilitate proactive participation in treatment and improving outcomes. It consists of a combination of effective psychosocial treatment programs that are very relevant to OT practice: motivational interviewing, self-monitoring, individualized visits, handicraft activities, individualized psychoeducation, and discharge planning. The IOT is summarized in Table 1. The IOT is provided on a one-on-one basis with occupational therapists in charge and is tailored to each participant. Therapeutic structure factors such as time, frequency, and place are set for everyone. The members of collaborative trial sites were received the training on the IOT before starting the trial.

**Table 1:** IOT program summary.

Program	Description
Motivational interviewing	• Regular implementation of motivational interviewing 2–3 times per week in 15–30 minute sessions • Intervention for improving motivational deficits • Promoting independence for OT by addressing the individual’s challenges while in hospital and after discharge
Self-monitoring	• Implementation on a weekly basis • Physical exercise on a one-on-one basis with an occupational therapist • Positive feedback for improving subjective experience deficits • Metacognitive training
Individualized visits	• Support strategy for carrying out activities of daily living away from the hospital room was implemented 2–3 times per week during the first half of the hospitalization • Support was provided, as necessary, for going out, utilizing social resources, and home visits prior to discharge in a community setting during the second half of the hospitalization
Handicraft activities	• Frequency was 3–5 times per week • Implementation time was about 30 minutes per session at the start of OT and was gradually extended to about 60 minutes • Utilization of constructive activities • Providing guidance on asking participants to attend to, concentrate on, precisely perform, and efficiently use instruments and materials • Bridging improvements in cognitive impairment and daily functioning
Individualized psychoeducation	• Illness management program and relapse prevention program were implemented 1–2 times per week for 45–60 minutes • Development of a crisis plan
Discharge planning	• Development of a post-discharge care plan and weekly action plan 1–2 times per week during the second half of the hospitalization • Skills training was implemented 1–2 times per week during the second half of the hospitalization

IOT: individualized occupational therapy; OT: occupational therapy.

Motivational interviewing [37] was regularly implemented 2–3 times per week in 15–30 minute sessions to improve motivational deficits [38] and enhance motivation for treatment. Intrinsic motivation is a factor that affects cognitive and social functioning [39–42]. In addition, learning and problem solving are improved, and social participation is promoted along with this improvement [43, 44]. Occupational therapists confirm the course of OT and its effects, and modify OT planning and goals. Furthermore, occupational therapists promote independence through OT by addressing individuals’ hospitalization and discharge challenges.

A self-monitoring program was implemented on a weekly basis to improve disturbances in self-body [45], subjective experience deficits [46], and metacognition [47–49]. Recovery from self-body disturbance is promoted through physical exercise, such as stretching, on a one-on-one basis with an occupational therapist. Positive feedback was provided to improve confidence, self-efficacy, and subjective experience deficits by addressing challenges and completing specific occupational activities. Subjective experience and metacognition in schizophrenia are moderators of cognitive and social functioning [50] and affect insight [51, 52]. Metacognitive training is implemented to improve cognitive insight [53, 54].

Individualized visits were used as a support strategy for conducting activities of daily living away from the hospital. It is possible that early OT will improve the functional independence of patients with acute schizophrenia [55]. During the first half of their hospitalization, this was implemented 2–3 times per week. During the second half of their hospitalization, support was provided as necessary in a community setting for going out, social resource utilization, and home visits prior to discharge.

The therapeutic use of handicraft activities is one feature of OT for schizophrenia. Constructive handicraft activities with clear procedures and good feasibility, such as Japanese paper collages, plastic models, Japanese paper crafts, and jigsaw puzzles, were used in the handicraft activities program. Handicraft activities were implemented 3–5 times per week. Implementation time was about 30 minutes per session at the start of OT and was gradually extended to about 60 minutes. To activate cognitive functioning such as vigilance, attention, executive function, and matching function, the patients were asked to attend to, concentrate on, precisely perform, and efficiently use the craft tools along with the individualized coaching. Occupational therapists observed the occupational performance characteristics of each patient. Interventions bridging the gap between improvements in cognitive impairment as observed in OT situations and daily functioning were implemented [56–58]. Bridging intervention help patients apply their cognition to daily functioning and promote socialization. Topics in this intervention included preparation for community living, the role of cognition in social skills, and problem solving concerning compensatory strategies for dealing with daily living challenges.

Psychoeducation for schizophrenia is effective for improving symptoms and insight and preventing relapse [59, 60]. Illness management and relapse prevention programs were implemented 1–2 times per week for 45–60 minutes each, and a crisis plan was developed as part of the individualized psychoeducation program. Occupational therapists supported each participant in identifying relapse signs and finding practical coping methods to address them [61]. A crisis plan was developed and shared with family members and other support persons through care conferences [62, 63].

Post-discharge care and weekly action plans, which included information on how to manage living time within a community setting, were developed to promote a smooth transition from the hospital setting to community living. These developments were conduct in collaboration with each patient 1–2 times per week during the second half of the hospitalization. Discharge planning is effective for preventing relapse and improving social functioning [64–66]. In addition, previous studies have reported the need to enhance functioning at discharge in treating schizophrenia [67, 68]. Skills training [69, 70], including training focused on living skills such as how to manage living time, money, and diet preparation. Interpersonal skills training focused on things such as how to greet people and ask for help if the need arose. These were both implemented to improve aspects of each participant’s daily functioning, such as instrumental daily living activities, education, and work with recovery states and each required 1–2 times per week during the second half of hospitalization.

#### GOT program

The GOT program is an activity-oriented group treatment program usually implemented based on a weekly OT schedule at each study site. The GOT includes the following programs: a group physical fitness program (stretching exercise, relaxation, and breathing); a group handicraft activities program, where participants choose and participate in desired activities programs; a group cooking program; a group music program (music appreciation and singing); a recreation program; and a group psychoeducation program. In the group handicraft activities program, each participant voluntarily completed the craft activities based on their preferences. The participants voluntarily selected any desired program among these and participated at an individualized rate. These programs were held either in hospital ward halls or OT rooms. Several patients simultaneously participated in each program and addressed activities in accordance with the program.

### Procedures

Referrals to the study were made by occupational therapists. Prospective participants met with occupational therapists of the study team who described the study procedures and, if the participant was interested, signed informed consent, and had a baseline assessment scheduled. Following completion of the baseline assessment, participants were randomized to either the GOT + IOT or the GOT alone groups by the project coordinator using a computer-generated randomization program. Randomization was stratified by sex (male/female), age (20–29, 30–39, 40–49, 50–59, and 60–65 years), and number of hospital stays (≤ 3 or ≥ 4). Within each stratum, patients were randomized 1:1 to the GOT + IOT or the GOT alone groups. Treatment assignment was not known in advance by study personnel. Post intervention assessment was conducted at discharge or 3 months following hospitalization (if the hospitalization period was greater than 3 months).

### Statistical analyses

The planned sample size for this study was 150 patients and 75 randomized patients per treatment group, with each patient contributing at least 1 baseline and 1 post-randomization assessments per treatment arm. This calculation was based on a two-sided test with σ = 2.286, σ x = 0.490, λ = 1.154, a type I error rate of 5, and 80% power to show statistically significant differences between treatments based on results of Shimada’s pilot, follow-up study of IOT [71]. The symbols used for the sample size calculation represented that σ was the standard deviation of the regression errors, σ x was the standard deviation of the independent variable, and λ was the slope of the linear regression line that we wish to detect. Result of the calculation demonstrated that the needed sample size was 130 patients and 65 patients per treatment group. However, an assumed patient dropout rate of approximately 20% led to a randomization target of 75 patients per treatment group. These calculations were also included because a further study is planned after this study to investigate the factors influencing rehospitalization after discharge with the same patient sample to examine whether IOT during hospitalization is effective for the prevention of rehospitalization.

Analyses on patients’ outcomes were conducted on an intention-to-treat basis. First, we compared the groups at baseline on demographics, cognition, and other measures using t-tests for the continuous variables and χ^2^ analyses for categorical variables. Second, feasibility and safety assessments were summarized using descriptive statistics. We computed the percentage of participants who were exposed to the IOT program, the average number of OT sessions, and the length of OT intervention. Third, to examine group differences in cognitive functioning outcomes, we included the BACS-J and the SCoRS-J over time (baseline and post assessment time) by treatment group (GOT + IOT and GOT only) using the linear mixed effects models with repeated measures: participants, site, and interaction of site by treatment as the random effect; age, sex, number of hospital stays, baseline scores, baseline IMI-J total score, treatment, assessment time points, and interaction of treatment by time as the fixed effects in the model. Moreover, we examined group differences in intrinsic motivation (IMI-J), social functioning (GAF), medication adherence (MMAS-8), and symptomatology (PANSS) over time (baseline and post assessment time) by treatment group (GOT + IOT and GOT only) using the linear mixed effects models with repeated measures: participants, site, and interaction of site by treatment as the random effect; age, sex, number of hospital stays, baseline scores, treatment, assessment time points, and interaction of treatment by time as the fixed effects in the model. Effect sizes (Cohen’s d) were calculated by dividing the differences of mean scores at post for groups by the estimated pooled standard deviation and defined as d ≥ 0.2 is small, d ≥ 0.5 is medium, and d ≥ 0.8 is large. The significant level was set at p < 0.05 for a two-sided test, and Bonferroni correction was applied to the statistical results to reduce type I errors generated by multiple comparisons. Statistical analyses were performed using JMP13.0.0 for Microsoft Windows (SAS Institute, Cary, NC, USA).

## Results

**Fig 1:**
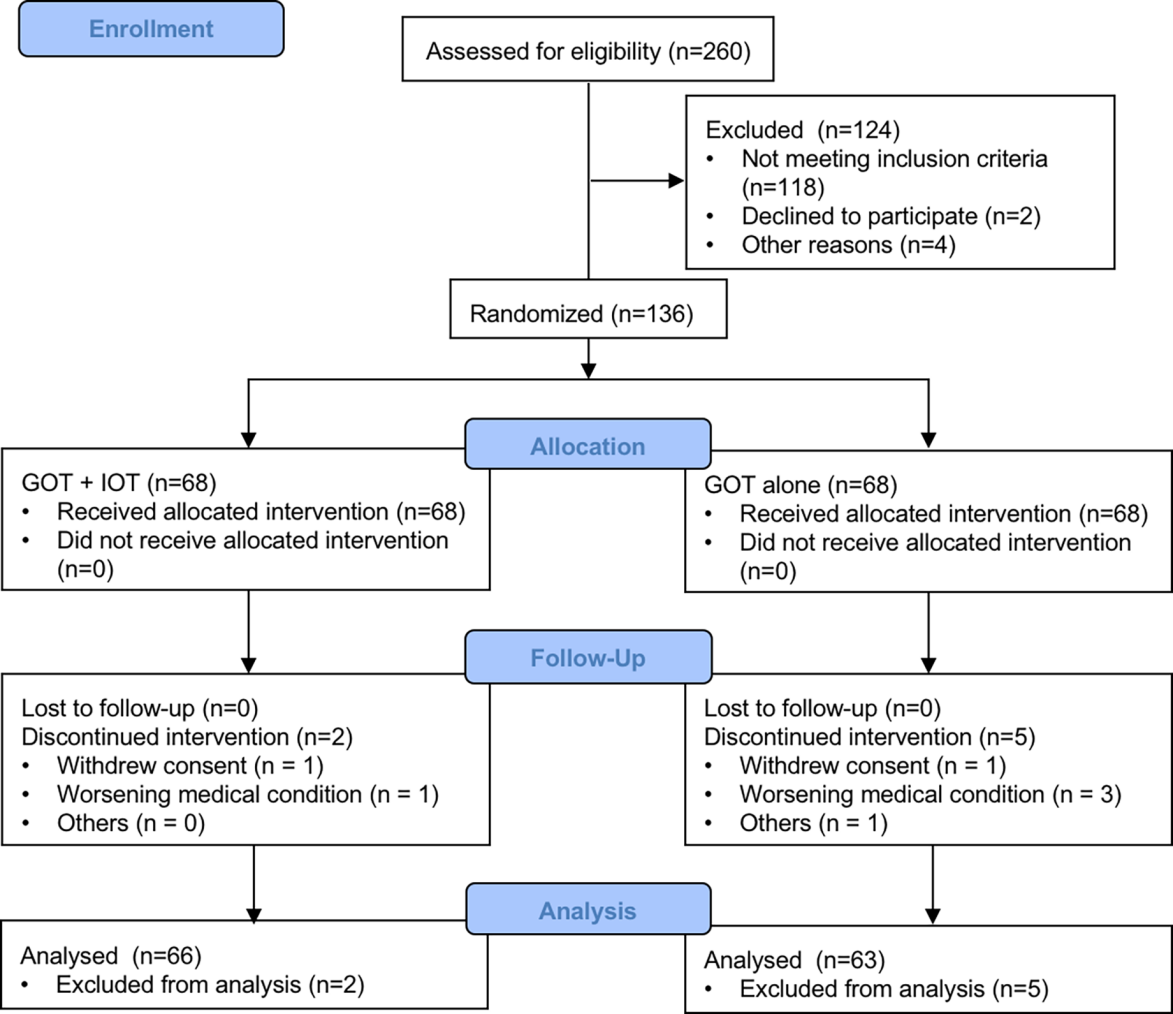
Patient disposition and study flow chart.

The patient disposition and study flow chart are described in Fig 1. Of the 260 patients who were assessed for eligibility in the study, 136 patients met criteria for study completion, 68 (50.00% of those randomized) in the GOT + IOT arm and 68 (50.00% of those randomized) in the GOT alone control arm. Of the 7 (5.15%) who dropped out at different points of the study, 2 (28.57% of those dropped out) were in the GOT + IOT condition and 5 (71.43% of those dropped out) were in the GOT alone control condition. There were a number of reasons including withdrew consent (n = 2), worsening a medical condition (n = 4), and other (n = 1). Comparison of participants who dropped out of the study and those who remained on demographics and clinical characteristics revealed no significant differences. The final sample used for analysis consisted of 129 patients, 66 (51.16%) in the GOT + IOT group and 63 (48.84%) in the GOT alone control group. All participants completed the baseline assessment, 129 (94.853%) completed the post assessment.

The demographics and clinical characteristics were comparable between treatment groups at baseline and are shown in Tables 2 and 3. Statistical tests comparing participants assigned to the GOT + IOT or the GOT alone groups indicated no significant differences in demographics and assessments results at baseline. All participants completed baseline assessment, 129 (94.85%) completed the discharge or 3-month assessment. 66 (97.06%) in the GOT + IOT group completed an average of 32.710 (SD = 7.920) OT sessions over an average of 69.935 (SD = 20.051) days and 63 (92.65%) in the GOT alone control condition group completed an average of 34.824 (SD = 7.350) OT sessions over an average of 73.441 (SD = 17.291) days, which did not differ significantly (number of OT sessions, t = 1.578, p = 0.117; length of OT intervention, t = 1.070, p = .287).

**Table 2:** Demographic characteristics and baseline assessments results by treatment group (GOT + IOT; GOT alone).

Variable	GOT + IOT (*n* = 68)		GOT alone (*n* = 68)		Statistic	*P*
Age (years), *mean (SD)*	41.391	(11.038)	43.389	(9.973)	*t* = 1.109	.269
Sex, *n* (%)						
• Male	34	(50.000)	33	(48.529)	*χ*^*2*^ = 0.0294	.864
• Female	34	(50.000)	35	(51.471)		
Diagnosis, *n* (%)						
• Schizophrenia	14	(20.588)	11	(16.176)	*χ*^*2*^ = 0.441	.507
• Schizoaffective disorder	54	(79.412)	57	(83.824)		
Age of onset (years), *mean (SD*)	22.019	(3.890)	23.300	(3.604)	*t* = 1.731	.087
Number of hospital stays (times), *mean (SD)*	3.688	(3.309)	5.944	(11.756)	*t* = 1.484	.140
Total length of hospital stays (months)^a^, *mean (SD)*	30.889	(43.068)	32.024	(43.561)	*t* = 0.149	.882
Education (year), *mean (SD*)	11.339	(1.958)	11.647	(2.100)	*t* = 0.863	.390
Experience of employment, *n* (%)						
• Yes	17	(25.000)	20	(29.412)	*χ*^*2*^ = 0.334	.563
• No	51	(75.000)	48	(70.588)		
Marital status, *n* (%)						
• Single	56	(82.353)	58	(85.294)	*χ*^*2*^ = 0.964	.810
• Married	7	(10.294)	4	(5.882)		
• Separated or divorced	4	(5.882)	5	(7.353)		
• Widowed	1	(1.471)	1	(1.471)		
Experience with OT, *n* (%)						
• Yes	28	(41.176)	30	(44.118)	*χ*^*2*^ = 0.120	.729
• No	40	(58.824)	38	(55.882)		
Length to OT from hospitalization (days)^b^, *mean (SD)*	12.613	(10.429)	9.574	(9.248)	*t* = 1.761	.081
Length of OT intervention (days)^c^, *mean (SD)*	69.935	(20.051)	73.441	(17.291)	*t* = 1.070	.287
Number of OT sessions (times), *mean (SD)*	32.710	(7.920)	34.824	(7.350)	*t* = 1.578	.117
Antipsychotic (mg/day)^d^, *mean (SD)*						
• Baseline	714.226	(242.970)	662.029	(250.817)	*t* = 1.203	.231
• Post	656.145	(254.030)	644.706	(248.549)	*t* = 0.259	.796
Prehospital social functioning^e^, *mean (SD)*						
• Withdrawal/social engagement	6.161	(2.327)	6.294	(2.023)	*t* = 0.348	.728
• Interpersonal communication	5.806	(1.836)	6.103	(1.846)	*t* = 0.917	.361
• Pro-social activities	14.613	(7.347)	16.471	(7.408)	*t* = 1.434	.154
• Recreation	18.968	(6.631)	20.309	(5.628)	*t* = 1.247	.215
• Independence-competence	21.516	(6.378)	20.235	(6.948)	*t* = 1.092	.277
• Independence-performance	17.516	(5.072)	16.368	(4.488)	*t* = 1.370	.173
• Employment/occupation	1.581	(2.526)	1.882	(2.011)	*t* = 0.757	.451
• SFS-J total	85.242	(21.760)	88.515	(19.031)	*t* = 0.915	.362

aThe total length of hospital stay represented the total length of all previous hospital stays.

bThe length to OT from hospitalization represented the length from hospitalization to start of OT.

cThe length of OT intervention represented the length from baseline to post intervention assessment.

dChlorpromazine equivalent dose

ePrehospital social functioning was assessed with SFS-J

GOT: group occupational therapy; IOT: individual occupational therapy; SD: standard deviation.

**Table 3:** Changes in effectiveness outcomes scores from baseline to post by treatment group (GOT + IOT; GOT alone) using linear mixed effects models repeated measures analyses.

Measure	Time	GOT + IOT (*n* = 66)		GOT alone (*n* = 63)			*F*		*Effect size*
		*Mean*	*(SD)*	*Mean*	*(SD)*	*Time*	*Group*	*Time × Group*	
BACS-J									
・Verbal memory	Baseline	-2.189	(1.327)	-2.442	(1.032)	94.822**	11.669	11.225**	0.58
	Post	-1.380	(1.161)	-2.053	(1.160)				
・Working memory	Baseline	-1.860	(1.043)	-1.700	(1.232)	58.084**	0.932	6.471*	0.28
	Post	-1.056	(0.962)	-1.374	(1.288)				
・Motor speed	Baseline	-3.431	(1.762)	-3.357	(1.793)	44.163**	0.081	0.253	0.10
	Post	-2.701	(1.479)	-2.854	(1.513)				
・Verbal fluency	Baseline	-1.326	(1.094)	-1.209	(0.984)	18.897**	6.606	21.099**	0.27
	Post	-0.932	(0.972)	-1.195	(0.961)				
・Attention	Baseline	-2.672	(1.275)	-2.549	(1.262)	109.574**	10.903	22.924**	0.30
	Post	-1.881	(1.055)	-2.216	(1.156)				
・Executive function	Baseline	-2.329	(1.931)	-2.524	(2.235)	58.102**	1.921	1.089	0.32
	Post	-1.098	(1.240)	-1.508	(1.328)				
・Composite score	Baseline	-2.306	(0.957)	-2.291	(0.977)	167.743**	12.721	14.160**	0.44
	Post	-1.507	(0.765)	-1.873	(0.885)				
SCoRS-J									
・SCoRS-J total	Baseline	55.468	(11.848)	55.794	(11.329)	90.788**	1.946	1.122	0.22
	Post	47.097	(9.516)	49.328	(10.713)				
・Patient global rating	Baseline	6.000	(1.882)	6.206	(1.873)	73.369**	7.882*	3.805	0.48
	Post	4.548	(1.646)	5.403	(1.947)				
・Interviewer global rating	Baseline	6.452	(1.434)	6.750	(1.262)	107.518**	2.778	0.236	0.30
	Post	5.290	(1.323)	5.702	(1.425)				
GAF									
・GAF score	Baseline	43.258	(10.823)	41.059	(10.676)	232.630**	0.849	0.702	0.36
	Post	54.000	(8.587)	50.493	(10.722)				
IMI-J									
・Interest/enjoyment	Baseline	24.677	(6.480)	25.191	(7.222)	97.030**	5.908	16.605**	0.55
	Post	32.000	(7.531)	28.030	(6.963)				
・Value/usefulness	Baseline	23.387	(8.279)	23.250	(8.357)	131.179**	3.526	10.673**	0.46
	Post	30.371	(7.549)	26.910	(7.633)				
・Perceived choice	Baseline	23.855	(7.135)	24.294	(6.907)	95.138**	9.151	19.124**	0.62
	Post	30.726	(6.611)	26.791	(6.044)				
・IMI-J total	Baseline	71.919	(18.494)	72.735	(20.777)	151.340**	7.792	21.773**	0.61
	Post	93.129	(18.229)	81.731	(18.870)				
MMAS-8									
・MMAS-8 score	Baseline	6.661	(1.270)	6.816	(1.234)	124.504**	1.516	8.458**	0.35
	Post	7.879	(1.161)	7.489	(1.070)				
PANSS									
・Positive	Baseline	26.694	(5.630)	28.588	(6.155)	108.170**	2.357	2.775	0.85
	Post	20.161	(5.442)	24.627	(5.054)				
・Negative	Baseline	26.032	(5.513)	25.809	(4.723)	114.074**	1.023	3.943	0.31
	Post	21.032	(5.141)	22.537	(4.450)				
・General psychopathology	Baseline	56.419	(11.845)	59.868	(12.450)	166.402**	0.710	0.262	0.52
	Post	46.000	(10.047)	51.134	(9.719)				
・PANSS total	Baseline	108.532	(20.049)	114.059	(20.367)	190.776**	0.822	1.761	0.59
	Post	87.613	(18.893)	98.149	(16.652)				

*p < 0.05

**p < 0.01.

GOT: group occupational therapy; IOT: individual occupational therapy; SD: standard deviation; BACS-J: Brief Assessment of Cognition in Schizophrenia Japanese version; SCoRS-J: the Schizophrenia Cognition Rating Scale Japanese version; SFS-J: the Social Functioning Scale Japanese version; GAF: the Global Assessment of Functioning scale; IMI-J: the Intrinsic Motivation Inventory Japanese version; MMAS-8: the Morisky Medication Adherence Scale-8; PANAS: the Positive and Negative Syndrome Scale.

The results of the linear mixed effects models examining treatment group differences over time on the outcomes for the participants who received IOT and those who did not are shown in Table 3. The linear mixed effects models examining the outcome measures yielded significant treatment by time interaction indicating that the effects of the treatment differed by group over time on verbal memory (F [1, 126] = 11.225, p < 0.01, Cohen’s d = 0.58), working memory (F [1, 126] = 6.471, p = 0.02, Cohen’s d = 0.28), verbal fluency (F [1, 126] = 21.099, p < 0.01, Cohen’s d = 0.27), attention (F [1, 126] = 22.924, p < 0.01, Cohen’s d = 0.30), and composite score (F [1, 126] = 14.160, p < 0.01, Cohen’s d = 0.44) in BACS-J; interest/enjoyment (F [1, 126] = 16.605, p < 0.01, Cohen’s d = 0.55), value/usefulness (F [1, 126] = 10.673, p < 0.01, Cohen’s d = 0.46), perceived choice (F [1, 126] = 19.124, p < 0.01, Cohen’s d = 0.62), and IMI-J total (F [1, 126] = 21.773, p < 0.01, Cohen’s d = 0.61) in IMI-J; MMAS-8 score (F [1, 126] = 8.458, p < 0.01, Cohen’s d = 0.35). Participants in the GOT + IOT demonstrated significant improvements in CSQ-8J compared with GOT only (t = 3.282, p < 0.01, Cohen’s d = 0.59).

## Discussion

This study examined the feasibility and effectiveness of conducting a multicenter, open-labeled, blinded-endpoint, randomized controlled trial using GOT + IOT group compared with a control condition of GOT alone group in the Japanese psychiatric hospital setting. This is the first randomized trial to demonstrate that the IOT program is feasible for use in Japanese psychiatric hospitals and can improve cognitive functioning and other outcomes in patients with schizophrenia.

The results of this study provide encouraging support for the feasibility of IOT for helping participants with schizophrenia who are enrolled in an OT program at Japanese psychiatric hospitals. The retention rate of the participants in this study was very high; 66 of 68 patients (97.06%) who agreed to participate were successfully engaged and completed the IOT arm from hospitalization to discharge or 3 months following hospitalization. This suggests the implementation of IOT did not have any adverse effects on patients with acute schizophrenia in the IOT program, and that IOT could be feasible for use in a Japanese psychiatric hospital setting.

The study also provides support for the effectiveness of the IOT in improving cognitive functioning (BACS-J) and other outcomes including intrinsic motivation (IMI-J), medication adherence (MMAS-8), and treatment satisfaction (CSQ-8J) in patients with schizophrenia. However, the results of this study may only suggest not so much the effectiveness of the IOT alone but the effectiveness of the adding IOT to GOT on outcomes. Although patients in the GOT alone group also showed the trend to improve several outcomes, the greater magnitude of outcomes improvements in the GOT + IOT were demonstrated compared with those in the GOT alone. For greater improving outcomes, it may be important to implement not only the IOT alone but also the adding IOT to GOT.

For the BACS-J, patients who participated in the GOT + IOT demonstrated significant improvements in several areas of cognitive functioning including verbal memory, working memory, verbal fluency, attention, and composite score compared with those who participated in the GOT alone. Improvements in these areas of functioning, and their magnitude, are like our previous pilot study [18], demonstrating the strength of IOT for improving cognitive impairment in patients with schizophrenia. In addition, these statistically significant improvements in BACS-J were noted to trend consistent with effect sizes, with outcome representing from small to medium effect sizes for GOT + IOT vs GOT alone groups in the BACS-J.

The IOT program involves multiple components including motivational interviewing, self-monitoring, individualized visits, handicraft activities, individualized psychoeducation, and discharge planning. The main goal of the IOT program was to integrate the enhancement of cognitive functioning and maximize functional outcomes for patients with schizophrenia. With this study design, it is not possible to clearly identify how the program works, or what components were essential; however, several conjectures may provide useful avenues for future study and clinical implications.

One important finding is that participants in the GOT + IOT improved in broad areas of cognitive functioning as measured by the BACS-J compared with the GOT alone. This is consistent with our previous study [18]. Several distinct factors could account for this finding. Although it is difficult to verify with this study design that handicraft activities could improve cognitive impairment, it was characteristic of IOT to use the handicraft activities for improving cognitive impairment of patients with schizophrenia. The use of handicraft activities with individualized coaching by occupational therapists is believed to contribute to improve cognitive impairment for patients with schizophrenia. Occupational performance focusing on aspects of cognitive functioning such as vigilance, attention, and matching function through the implementation of handicraft activities may activate patients’ brain function. In addition, the involvement of occupational therapists in promoting these cognitive activities may enhance cognitive impairment improvements.

Furthermore, bridging interventions were implemented in the IOT program. These may have promoted independence within the OT program, thus improving intrinsic motivation and metacognition. Previous studies have reported that intrinsic motivation and metacognition are factors that affect cognitive functioning [40, 48, 50]. Promoting these improvements through motivational interviewing and self-monitoring program may have led to cognitive impairment improvements of greater magnitude for participants in the GOT + IOT compared with those in the GOT alone group.

Although the linear mixed effects models indicated nonsignificant interaction of treatment by time on PANSS, there were significant main effects of time on each subscale and total score in PANSS, with both groups showing improved symptoms over time. It is important to note that symptom scores as measured by PANSS did not worsen in the GOT + IOT group even though patients were more involved in intensive individualized treatment as IOT. Increased intensive individualized treatment demands may increase stress and have a possibility to worsen symptoms. Better functional status in the context of acute treatment for schizophrenia translates to a positive outcome for patients in the GOT + IOT. However, there was no evidence that GOT + IOT improved symptom in patients with schizophrenia. Although there were not statistically significant differences in symptoms as measured by PANSS across treatment groups in this study, effect sizes of the change between groups were observed from small to large in the each domain including positive symptom, negative symptom, general psychopathology, and total score in PANSS. Our previous study of GOT + IOT demonstrated significant improvements in positive subscale, general psychopathology subscale, and total score in PANSS [18], and other previous studies reported that OT had beneficial effects on symptoms of patients with schizophrenia [16, 17]. More studies are needed to evaluate the impact of OT on symptom of schizophrenia.

Interventions bridging the gap between improvements of cognition and daily functioning and discharge planning were implemented to improve social functioning in the IOT program. However, participants in the GOT + IOT group did not show significant improvements regarding the factors related to social functioning measured by the GAF. One of the reasons for this may be that this study’s interventions were implemented as part of hospital treatment. It is recommended that skills training for improving social functioning and learning daily living skills be enacted within a community setting [70, 72]. Therefore, it is necessary to implement interventions for improving social functioning at not only a hospital, but also a community treatment setting after discharge.

A number of study limitations should be noted. First, considering that cognitive functioning and other outcomes were assessed at only 3 months following participation in the OT program, there is a need to evaluate the long-term effects of IOT during hospitalization. Second, many of the study participants were only short-term hospitalized patients with acute schizophrenia; therefore, interventions to improve cognitive functioning and other outcome domains for long-term hospitalized patients were not performed. Third, the number of OT sessions per subprogram in IOT was not measured; therefore, it is difficult to clearly identify how the program works, or what components are essential. Fourth, the optimal dose such as time and frequency of OT implementation for each the GOT and IOT to maximize outcome was not examined, this will require further study. Despite these limitations, this study provides staunch support for the feasibility of implementing IOT and the benefits of IOT for improving cognitive functioning and other outcomes.

To summarize, this study provides further evidence for the feasibility of IOT at Japanese psychiatric hospitals and the effectiveness of providing IOT in addition to GOT for improving the cognitive functioning and other outcomes of patients with schizophrenia or schizoaffective disorder. These findings encourage the growth of IOT among psychiatric hospitals and have the potential to improve psychosocial treatment in Japan. It is important to examine that the impact of IOT on relapse and rehospitalization after this study, because the relapse and rehospitalization are frequent among patients with schizophrenia during the clinical course and worsens their functional outcomes [73–75]. Future study is warranted to address the prognostic significance of IOT by exploring the impact of IOT and clinical variables on functional outcome.

## Supporting information

S1 File CONSORT checklist. (DOCX)

S2 File Protocol in Japanese. (DOCX)

S3 File Protocol in English. (DOCX)
