# Correction: A multicenter, randomized controlled trial of individualized occupational therapy for patients with schizophrenia in Japan

**DOI:** 10.1371/journal.pone.0205549

**Published:** 2018-10-09

**Authors:** Takeshi Shimada, Manami Ohori, Yusuke Inagaki, Yuko Shimooka, Naoya Sugimura, Ikuyo Ishihara, Tomotaka Yoshida, Masayoshi Kobayashi

The following copyright and trademark right statement is missing from the Acknowledgments and Table 3 footnote: Use of the ©MMAS is protected by US copyright laws. Permission for use is required. A license agreement is available from Donald E. Morisky, 294 Lindura Court, Las Vegas, NV 89138–4632; dmorisky@gmail.com [2–4].

Three citations are missing from S3 File. Please see the corrected S3 File below.

There is an error in Table 2, as the number of participants and the descriptions of schizophrenia and schizoaffective disorder in the diagnostic item of Table 2 was reversed. Specifically, the numbers in rows 7 and 8 were swapped. Please see the corrected Table 2 here.

**Table 2:** Demographic characteristics and baseline assessments results by treatment group (GOT + IOT; GOT alone).

Variable	GOT + IOT (*n* = 68)		GOT alone (*n* = 68)		Statistic	*P*
Age (years), *mean (SD)*	41.391	(11.038)	43.389	(9.973)	*t* = 1.109	.269
Sex, *n* (%)						
• Male	34	(50.000)	33	(48.529)	*χ*^*2*^ = 0.0294	.864
• Female	34	(50.000)	35	(51.471)		
Diagnosis, *n* (%)						
• Schizophrenia	54	(79.412)	57	(83.824)	*χ*^*2*^ = 0.441	.507
• Schizoaffective disorder	14	(20.588)	11	(16.176)		
Age of onset (years), *mean (SD*)	22.019	(3.890)	23.300	(3.604)	*t* = 1.731	.087
Number of hospital stays (times), *mean (SD)*	3.688	(3.309)	5.944	(11.756)	*t* = 1.484	.140
Total length of hospital stays (months)^a^, *mean (SD)*	30.889	(43.068)	32.024	(43.561)	*t* = 0.149	.882
Education (year), *mean (SD*)	11.339	(1.958)	11.647	(2.100)	*t* = 0.863	.390
Experience of employment, *n* (%)						
• Yes	17	(25.000)	20	(29.412)	*χ*^*2*^ = 0.334	.563
• No	51	(75.000)	48	(70.588)		
Marital status, *n* (%)						
• Single	56	(82.353)	58	(85.294)	*χ*^*2*^ = 0.964	.810
• Married	7	(10.294)	4	(5.882)		
• Separated or divorced	4	(5.882)	5	(7.353)		
• Widowed	1	(1.471)	1	(1.471)		
Experience with OT, *n* (%)						
• Yes	28	(41.176)	30	(44.118)	*χ*^*2*^ = 0.120	.729
• No	40	(58.824)	38	(55.882)		
Length to OT from hospitalization (days)^b^, *mean (SD)*	12.613	(10.429)	9.574	(9.248)	*t* = 1.761	.081
Length of OT intervention (days)^c^, *mean (SD)*	69.935	(20.051)	73.441	(17.291)	*t* = 1.070	.287
Number of OT sessions (times), *mean (SD)*	32.710	(7.920)	34.824	(7.350)	*t* = 1.578	.117
Antipsychotic (mg/day)^d^, *mean (SD)*						
• Baseline	714.226	(242.970)	662.029	(250.817)	*t* = 1.203	.231
• Post	656.145	(254.030)	644.706	(248.549)	*t* = 0.259	.796
Prehospital social functioning^e^, *mean (SD)*						
• Withdrawal/social engagement	6.161	(2.327)	6.294	(2.023)	*t* = 0.348	.728
• Interpersonal communication	5.806	(1.836)	6.103	(1.846)	*t* = 0.917	.361
• Pro-social activities	14.613	(7.347)	16.471	(7.408)	*t* = 1.434	.154
• Recreation	18.968	(6.631)	20.309	(5.628)	*t* = 1.247	.215
• Independence-competence	21.516	(6.378)	20.235	(6.948)	*t* = 1.092	.277
• Independence-performance	17.516	(5.072)	16.368	(4.488)	*t* = 1.370	.173
• Employment/occupation	1.581	(2.526)	1.882	(2.011)	*t* = 0.757	.451
• SFS-J total	85.242	(21.760)	88.515	(19.031)	*t* = 0.915	.362

^a^The total length of hospital stay represented the total length of all previous hospital stays.

^b^The length to OT from hospitalization represented the length from hospitalization to start of OT.

^c^The length of OT intervention represented the length from baseline to post intervention assessment.

^d^Chlorpromazine equivalent dose

^e^Prehospital social functioning was assessed with SFS-J

GOT: group occupational therapy; IOT: individual occupational therapy; SD: standard deviation.

## Supporting information

S3 File Protocol in English. (DOCX)
