# Supplementary material for: A multicenter, randomized controlled trial of individualized occupational therapy for patients with schizophrenia in Japan
Source: PLoS One. 2018 Apr 5;13(4):e0193869. doi: 10.1371/journal.pone.0193869 (PMC5886394; doi:10.1371/journal.pone.0193869)
Supplement: S2 File — (DOCX) [file pone.0193869.s002.docx]

**自主臨床研究**

**統合失調症に対する個別作業療法の効果**

**多施設共同ランダム化比較試験**

**研　究　実　施　計　画　書**

研究責任者　　信州大学医学部　保健学科　小林　正義

2015年9月1日　計画書案　第1版作成

目次

0.　概要

　0.1.　シェーマ

　0.2.　目的

　0.3.　対象

　0.4.　目標登録症例数と試験期間

　0.5.　研究デザイン

　0.6.　評価項目

　0.7.　問い合わせ先

1.　目的

2.　背景と根拠

3.　適格性の基準

　3.1.　選択基準

　3.2.　除外基準

4.　研究の方法

　4.1.　研究デザイン

　4.2.　研究のアウトライン

　4.3.　介入の方法

　4.4.　症例登録・割付の方法

　　4.4.1.　症例登録

　　4.4.2.　割付方法と割付調整因子

　4.5.　研究終了後の対応

5.　評価項目

　5.1.　主要評価項目

　5.2.　副次的評価項目

6.　有害事象の取り扱い

　6.1.　有害事象の定義

　　6.1.1.　有害事象の定義

　　6.1.2.　重篤な有害事象の定義

　6.2.　有害事象発現時の被験者への対応

　6.3.　有害事象の評価・報告

　6.4.　予測される有害事象

7.　目標登録症例数

8.　統計的事項

　8.1.　目標登録症例数の設定根拠

　8.2.　統計解析方法

　8.3.　解析項目・方法

　　8.3.1.　解析対象の概要

　　8.3.2.　主要評価項目及び副次評価項目に関する仮説検証的解析

　　8.3.3.　主要評価項目及び副次評価項目に関する仮説探索的解析

9.　モニタリング

10.　倫理的事項

　10.1.　遵守すべき諸規則

　10.2.　インフォームド・コンセント

　10.3.　個人情報の保護

11.　研究実施計画書等の変更

12.　研究の費用

　12.1.　研究資金及び利益相反

　12.2.　被験者の費用負担

　12.3.　健康被害への対応と補償

13.　研究機関と研究の終了・早期中止

　13.1.　研究期間

　13.2.　研究の終了

　13.3.　研究の早期中止

14.　医療機器等の保存及び使用方法並びに保存期間

15.　記録の保存

16.　研究の公表と成果の帰属

　16.1.　臨床試験登録

　16.2.　成果の帰属

17.　研究実施体制

18.　参考資料・参考文献

19.　付録

0.　概要

0.1.　シェーマ

主な適格基準

統合失調症または統合失調感情障害の新規入院患者

登録・割付け

目標登録症例数：150例（介入群75例、対照群75例）

症例登録期間：2015年11月2日～2017年3月31日

割付調整因子：年齢、性別、入院回数

介入群

個別作業療法

+

集団作業療法

対照群

集団作業療法

認知機能障害に対する効果判定：退院時（または入院3ヵ月後）

介入群

対照群

再入院率の比較、再入院に関わる要因の検討：退院2年後

0.2.　目的

　1）統合失調症の認知機能障害に対する個別作業療法の効果を、集団作業療法との比較によって評価する。

　2）統合失調症患者の再入院に関わる要因を、入院中の作業療法の種別（個別作業療法と集団作業療法）を変数に加えて、探索的に検討する。

0.3.　対象

　統合失調症または統合失調感情障害と診断され精神科病院（病棟）に新規に入院した患者。

0.4.　目標登録症例数と試験期間

目標登録症例数：150名（介入群75例、対照群75例）

症例登録期間：2015年11月2日～2017年3月31日

試験実施期間：2015年11月2日～2019年3月31日

0.5.　研究デザイン

- デザインの特徴：多施設共同によるランダム化比較試験
- 対照の種類：通常治療対照（集団作業療法）
- ランダム化：性別、年齢、入院回数で層別化した後に最小化法によるランダム割付
- 盲検化のレベル：担当作業療法士と主治医以外の関係者（看護師、臨床心理士、精神保健福祉士、地域スタッフ等）および評価者には割付を盲検化

0.6.　評価項目

主要評価項目：介入前後の認知機能検査の成績。

副次評価項目：介入前後の精神症状と社会機能検査の成績、退院時の治療満足度、退院2年後の再入院率。

0.7.　問い合わせ先

【研究責任者】

小林　正義　　信州大学大学院医学系研究科保健学専攻　教授

　　　　　　　〒390-8621　松本市旭3-1-1

TEL/FAX：0263-37-2403、E-mail：mkobaya@shinshu-u.ac.jp

【試験内容に関する問い合わせ】

研究事務局：医療法人清泰会メンタルサポートそよかぜ病院

島田　　岳　　信州大学大学院医学系研究科保健学専攻博士後期課程　大学院生

　　　　　　　医療法人清泰会メンタルサポートそよかぜ病院　作業療法士

　〒386-0401　長野県上田市塩川3057-1

　　　　　　　TEL：0268-35-0305、FAX：0268-35-0534、E-mail：ot@seitaikai.co.jp

1.　目的

　1）統合失調症の認知機能障害に対する個別作業療法の効果を、集団作業療法との比較によって評価する。

　2）統合失調症患者の再入院に関わる要因を、入院中の作業療法の種別（個別作業療法と集団作業療法）を変数に加えて探索的に検討する。

2.　背景と根拠

　日本の精神病床（339,780床）はOECD加盟国のうち最も多い^1-3）^。また、日本の精神病床は9割が民間精神科病院で占められ^4）^、入院患者の平均在院日数（296日）はOECD加盟国のなかで最も長い^1-3）^。このような状況を改善するため、現在、日本の精神科医療では、早期治療・リハビリテーションによる退院促進、退院後の地域生活支援の充実が重要課題となっている。

　精神科病院の入院患者は統合失調症が6割を占めており最も多い^3）^。統合失調症の入院治療では、急性期症状を薬物療法と良質な休息によって沈静化し、早期リハビリテーションによって中核障害である認知機能障害^5-9）^を改善させ、社会機能の向上と再発・再入院の予防を図ることが重要である。

　早期リハビリテーションとして多くの入院患者に作業療法（Occupational Therapy：OT）が実施されている。OTでは、個々の回復状態や生活課題を考慮した個別支援が必要である。しかし、現行の診療報酬体系では、「1日当たりの取扱い患者数は、概ね25人を1単位として、1人の作業療法士の取扱い患者数は1日2単位50人以内を標準とする」とされているため、採算性が求められる民間精神科病院では個別支援が実施しにくいという問題がある。この現行規程は、1974（昭和49）年に長期入院患者に対する集団的処遇を想定して策定されたもので、短期入院患者への個別支援を必要とする現代の精神科リハビリテーションの実情にはそぐわない。診療報酬の改正が望まれるが、現状では個別対応を基本とするOT（以下、個別OT）の有効性は検証されておらず、臨床では従来の集団的処遇（以下、集団OT）を基本に、必要に応じて一部の患者に部分的に個別OTが提供されているに過ぎない。

　我々が実施したパイロット研究では、従来の集団OTに個別OTを加えた群では、集団OTのみを実施した群に比べて、退院時または入院3ヵ月後の認知機能障害の改善が有意に大きく^10）^、さらに退院後1年間の再発（再入院）が少なかった^11）^。これらの結果は個別OTの有効性を示唆するが、結果は単一施設の所見であり、さらに個別OTの実施については患者が任意に選択したため、結果に選択バイアスが影響している可能性は否定できない。

　そこで本研究では、対象患者を、集団OTに加えて個別OTを実施する群と、従来の集団OTのみを実施する群とにランダムに割り付ける比較試験を多施設共同で実施し、統合失調症の認知機能障害に対する個別OTの効果を検証する。また、退院した患者の追跡調査を実施し、2年後の再入院（再発）率を比較するとともに、再入院に関わる要因を探索する。

3.　適格性の基準

3.1.　選択基準

　以下の基準を全て満たす患者を対象とする。

①精神科病院または精神科病床への新規入院患者。

②DSM-IV-TR^12）^の診断基準により統合失調症または統合失調感情障害と診断された者。

③主治医よりOTの実施が指示された者。

④本研究への参加にあたり十分な説明を受けた後、患者本人の自由意思に基づき文書による同意が得られた患者。

3.2.　除外基準

以下の除外基準のいずれにも該当しない患者を対象とする。

①20歳未満または65歳以上の者。

②知的障害、アルコール・薬物依存（物質乱用・依存）、認知症、てんかん等の合併のある者。

③頭部外傷、脳血管疾患等の脳器質疾患のある者。

④認知機能検査の実施が困難な者。

⑤身体機能障害等により個別の介入が必要な者。

⑥本研究への参加にあたり十分な説明を受けた後、患者本人の自由意思に基づき文書による同意が得られなかった患者。

⑦患者の主治医または研究責任者が被験者として不適当と判断した患者。

4.　研究の方法

4.1.　研究デザイン

- デザインの特徴：多施設共同によるランダム化比較試験
- 対照の種類：通常治療対照（集団OT）
- ランダム化：性別、年齢、入院回数で層別化した後に最小化法によるランダム割付
- 盲検化のレベル：担当作業療法士と主治医以外の関係者（看護師、臨床心理士、精神保健福祉士、地域スタッフ等）及び評価者には割付を盲検化

4.2.　研究のアウトライン

　精神科病院（病棟）に入院し、DSM-IV-TRの診断基準により統合失調症または統合失調感情障害と診断された者のうち、医師よりOTの指示があり、研究参加への同意が得られた患者を対象とする。対象者を、従来の集団OTに加えて個別OTを実施する群（個別OT+集団OT：介入群）と従来の集団OTを実施する群（集団OTのみ：対照群）にランダムに割り付け、認知機能障害に対する個別OTの効果を検証する。同意取得後に事前（ベースライン）評価を行い、対象者を介入群と対照群にランダムに割付け、それぞれの介入を行う。退院時（または入院3ヵ月後）に事後評価を行い群間比較する。さらに、入院1年以内に退院した患者を対象に追跡調査を行い、2年後の再入院（再発）率を群間比較するとともに、再入院に関わる要因を入院中のOTの種別（個別OTと集団OT）を変数に加えて探索的に検討する。

図1　研究のアウトライン

4.3.　介入の方法

　介入群には各施設が行っている通常のOTプログラム（集団OT）に加えて、表1に示す個別OTプログラムを実施する。個別OTプログラムは、1）個別面接、2）セルフモニタリング、3）外出・訪問指導、4）個別作業指導、5）個別心理教育^注2）^、6）退院時指導からなる。介入群では、これらのプログラムを作業療法士が対象者と1対1の時間を設定して実施する。実施にあたっては、チェックリスト^注1）^とマニュアル^16）^を使用する。また、研究を開始するにあたっては、あらかじめ研究協力者（担当作業療法士）を集めて個別OTプログラムの実施方法に関する研修会を開催し、介入方法の均一性を図る。

　対照群では、各施設で行われている従来のOTプログラム（集団OT）のみを行う。OTの実施時間は介入群・対照群ともに1回につき1～2時間、週3～5回を目安に、対象者の回復状態に応じて加減する。なお、介入群ではOT時間の半分以上を個別OTのプログラムに当てる。

表1　個別OTのプログラム

| プログラム | 実施内容 |
| --- | --- |
| 1）個別面接 | 週に2～3回の頻度で個別面接を実施し、リハビリテーション計画と、これまでの改善点と今後の課題を整理する。 |
| 2）セルフモニタリング | チェックリスト^注1）^を用いて個々の主観的な体験（身体感覚、疲労感、回復・改善感など）のメタ認知を促す。 |
| 3）外出・訪問指導 | 入院早期より病室訪問による離床支援と活動支援を行う。必要に応じて外出指導、退院前訪問指導を実施する。 |
| 4）個別作業指導 | 説明図に沿って行う構成的作業を利用する。作業遂行の正確性、道具や材料の効率的使用法を指導し、注意機能、照合機能の促進を図る。 |
| 5）個別心理教育^注2）^ | 疾病教育、再発サインの検討、再発予防プログラムの作成、クライシス・プランを作成する。 |
| 6）退院時指導 | 経過の振り返り、退院後のケアプラン^注2）^・活動スケジュールを作成する。 |

注1）：チェックリストは、入院生活チェックリスト（ISDA）^13)^と気分と疲労のチェックリスト（SMSF）^14, 15)^を使用する。

注2）：「個別心理教育」と「退院後のケアプラン」の作成は、日本作業療法士協会編「作業療法マニュアル41、精神障害の急性期作業療法と退院促進プログラム」^16)^を参考に実施する。

4.4.　割付方法と割付調整因子

　対象患者は介入群または対照群にランダムに割り付けられる。ランダム化の方法は性別と年齢で層別化した後に最小化法を用い、割付調整因子は年齢（10歳毎の各年代）、性別（男：女）、入院回数（4回未満・4回以上）とする。

4.5.　研究終了後の対応

　本研究終了後は、この研究で得られた成果も含めて、研究責任者は被験者に対し最も適切と考えられる医療を提供する。

5.　評価項目（エンドポイント）

5.1.　主要評価項目

　認知機能の介入前後の変化

5.2.　副次評価項目

　社会機能と精神症状（陽性症状と陰性症状）の介入前後の変化、治療満足度、退院2年間の再入院率（退院から1ヵ月間毎に各治療群別に再入院率を算出）

5.3.　観察・検査項目

- 介入前後の認知機能検査には、統合失調症認知機能簡易評価尺度日本語版（BACS-J）^17, 18)^と統合失調症認知評価尺度日本語版（SCoRS-J）^19, 20)^を使用する。また、社会機能の評価には社会機能評価尺度日本語版（SFS-J）^21, 22)^を使用する。
- BACS、SCoRS、SFSはいずれも米国の大規模プロジェクト（the National Institute of Mental Health Measurement and Treatment Research to Improve Cognition in Schizophrenia: NIMH-MATRICS）で検討され推奨されている尺度である。本研究ではこれらの日本語版であるBACS-J、SCoRS-J、SFS-Jを使用する。なお、SCoRS-Jには患者用フォームの他に、介護者（家族）用と評価者用のフォームがあるが、本研究では患者用フォームを使用する。
- 内発的動機付けの評価には内発的動機付け尺度日本語版（IMI-J）^23）^を使用する。
- 精神症状の評価には陽性・陰性症状評価尺度（PANSS）^24)^と機能の全体的評定尺度（GAF）^25)^を使用する。
- 退院時の治療満足度の評価には日本語版CSQ8（CSQ-8J）^26, 27)^を使用する。
- 再入院の有無と、再入院した場合には退院から再入院までの日数を観察する。

6.　有害事象の取り扱い

6.1.　有害事象の定義

6.1.1.　有害事象の定義

　有害事象（AE：Adverse Event）とは、OTを受けた被験者に生じたあらゆる好ましくない医療上の出来事をいい、OTとの因果関係の有無は問わない。

6.1.2.　重篤な有害事象の定義

　重篤な有害事象（SAE：Severe Adverse Event）とは、有害事象のうち以下に該当するものをいう。

　1．死亡

　2．死亡につながるおそれのあるもの

　3．治療のための入院または入院期間の延長が必要となるもの

　4．障害

　5．障害につながるおそれのあるもの

　6．その他1～5に準じて重篤であるもの

　7．後世代における先天性の疾病または異常をきたすもの

6.2.　有害事象発現時の被験者への対応

　本研究は、既に精神科病院で実施されているOTを、個別に、より丁寧に実践し、従来の集団OTとの効果の違いを明らかにするもので、これによって有害事象が生じることは考えにくい。しかし、研究責任者または研究分担者は、有害事象を認めたときは、直ちに適切な処置を行うとともに、診療録ならびに症例報告書にその旨を記載する。また、個別OTを中止する場合や、有害事象に対する治療が必要となった場合には、被験者にその旨を伝える。

6.3.　有害事象の評価・報告

　研究担当者は、研究期間中に発現した全ての重篤な有害事象ならびに研究終了（中止）後2日以内に発現したOTとの関連性が疑われる重篤な有害事象について、速やかに医学部長に報告する。報告は、「重篤な有害事象に関する報告手順」に従い、発生後速やかに第一報報告（緊急報告）を行い、原則として7日以内に第二報報告（詳細報告）を行う。また、必要に応じて、第三報以降の報告も行う。その他の有害事象については、研究担当者は適切に診療録および症例報告書に記載する。

6.4.　予測される有害事象

　統合失調症の急性期などでは、個別OTが過度な賦活刺激として作用し、不安感や疲労感などが増す可能性がある。

7.　目標登録症例数

　目標登録症例数は150例（介入群75例、対照群75例）、信州大学0例とする。

8.　統計的事項

8.1.　目標登録症例数の設定根拠

　Shimada et al（2015）のパイロット研究^11）^を参考に、Department of Biostatistics, Vanderbilt University School of Medicineが提供している「Power and Sample Size Calculation」（PS）を用いて、目標症例数を算出する。σ=2.286、σ_x_=0.490、λ=1.154、有意水準α error=0.05、β error=0.20とすると、最低限の必要サンプル数は、130名となる。脱落例等を考慮して、目標登録症例数は150名とした。

8.2.　統計解析方法

　解析は治療企図解析（Intention-to-treat analysis：ITT解析）を行う。解析対象集団の定義は（1）選択基準から逸脱している場合、（2）除外基準に抵触している場合のいずれかに該当する被験者を除いて構成される集団とする。

8.3.　解析項目・方法

　以下に統計解析の概要を示す。検定の有意水準は両側0.05とする。統計解析責任者は統計解析計画書を別途作成し、解析方法の詳細を規定する。

8.3.1.　解析対象の概要

1）解析対象集団の構成

　登録症例数、適格症例数、治療開始症例数、解析対象症例数を治療群別に算出する。

　OTを実施しなかった症例及び登録後に不適格が判明した症例については、理由別に集計する。

2）被験者背景因子及びベースラインデータ

　被験者背景因子及びベースラインデータについて、適切な要約統計量を治療群別に算出する。

3）治療情報

　治療（OT）の実施方法について、適切な介入方法を治療群別に記載する。

　試験中止の有無及びその理由について治療群別に集計する。

8.3.2.　主要評価項目及び副次評価項目に関する仮説検証的解析

　以下の手順で解析する。中止・脱落例はその理由を明らかにしてデータを集積する。

①ベースライン時（割付前）に、BACS-J、SCoRS-J、SFS-J、PANSS、GAFについて、対応のないt検定、χ^2^検定を行う。

②退院時（退院が入院3ヵ月以上の場合には入院3ヵ月後）に、OTの効果を検討するために、各評価尺度、抗精神病薬の服薬量（CP換算）について、群（介入群、対照群）と時期（ベースライン時、退院時）を独立変数とした反復測定二元配置分散分析を行う。効果量（effect size）の指標はη^2^を用いる。

③CSQ-8Jは治療満足度を検討するために退院時（退院が入院3ヵ月以上の場合には入院3ヵ月後）に、対応のないt検定を行う。入院3ヵ月時の退院者数（退院率）は介入群と対照群の入院期間への影響の違いを検討するために、入院3ヵ月時にχ^2^検定を行う。

④個別OTの再入院の予防に対する効果を検討するために、追跡調査により退院後から1ヵ月毎に介入群と対照群の再入院率（再入院患者数）の算出とχ^2^検定を行う。

8.3.3.　主要評価項目及び副次評価項目に関する仮説探索的解析

　以下の手順で解析する。中止・脱落例はその理由を明らかにしてデータを集積する。

①退院後から2年間の再入院の有無を調査し、地域生活を継続していた者を安定群、再入院した者を再入院群とし割り付ける。被験者背景、ベースライン時の評価尺度スコア、退院時（または入院3ヵ月後）の評価尺度スコア、退院後の生活状況、外来治療の内容、地域保健福祉サービスについて、対応のないt検定、χ^2^検定を用いて安定群と再入院群についての単変量解析を行う。次に、再入院に関わる要因を検討するために、安定群と再入院群の単変量解析で有意差がみられた項目を独立変数、再入院の有無を従属変数とした二項ロジスティック回帰分析を行う。なお、独立変数の選択にあたっては、多重共線性の影響を考慮し、各項目間でPearsonの相関係数を算出する。

9.　モニタリング

　モニタリング担当者（島田岳：医療法人清泰会メンタルサポートそよかぜ病院 作業療法士）はモニタリング計画書を作成し、計画書に沿って定期的（1ヵ月毎）に問題点を種類別に要約し、試験モニタリング報告書としてまとめ、研究責任者に提出する。研究責任者は報告内容およびその詳細を確認し、問題の再発が危惧される場合には、注意喚起文書の発行や研究実施計画書の改訂等必要な処置を講ずる。

【モニタリング項目】

1）適格基準との整合性

2）治療計画との整合性

3）重篤な有害事象の未報告

4）発生した有害事象と中止基準との整合性

5）必須検査・観察項目の未実施

6）その他、プロトコール逸脱等の問題点

10.　倫理的事項

10.1.　遵守すべき諸規則

　本研究の関係者は「世界医師会ヘルシンキ宣言」および「人を対象とする医学系研究に関する倫理指針」を遵守する。

10.2.　インフォームド・コンセント

　研究担当者および研究協力者は、医学部医倫理委員会で承認の得られた同意説明文書を被験者に渡し、文書および口頭による十分な説明を行い、被験者の自由意思による同意を文書で取得する。

　研究担当者および研究協力者は、被験者の同意に影響を及ぼす情報が得られたときや、被験者の同意に影響を及ぼすような実施計画等の変更が行われるときは、速やかに被験者に情報提供し、研究に参加するか否かについて被験者の意思を予め確認するとともに、事前に医学部医倫理委員会の承認を得て同意説明文書等の改訂を行い、被験者の再同意を得ることとする。

　なお、同意説明文書は、以下の内容を含むものとする。

1）研究への参加は任意であること、同意しなくても不利益を受けないこと、同意は撤回できること

2）研究の意義（背景）、目的、対象、方法、実施期間、予定被験者数

3）研究に参加することにより期待される利益、起こりえる不利益

4）個人情報を含めた試料等の取扱い、保存期間と廃棄方法、研究方法等の閲覧

5）研究成果の発表および特許が発生した場合の取扱い

6）研究に係る被験者の費用負担、研究資金源と利益相反

7）研究の組織体制、研究に関する問い合わせ、苦情等の相談窓口（連絡先）

8）被験者に健康被害が発生した場合の対応と補償の有無

10.3.　個人情報の保護

　研究実施に係る試料等を取扱う際は、この研究に直接関与しない個人情報管理者が被験者の個人情報とは無関係の番号を付して管理し、被験者の秘密保護に十分配慮する。試料等を研究者が分析する場合はこの番号を使用し、被験者の個人情報が外部に漏れないよう十分配慮する。また、各研究協力施設の個人情報の保護は担当作業療法士が行い、研究事務局には個人情報を削除したデータのみを送付する。研究の結果を公表する際は、被験者を特定できる情報を含まないようにする。研究の目的以外に、研究で得られた被験者の試料等を使用しない。

11.　研究実施計画書等の変更

　本研究の研究実施計画書や同意説明文書の変更または改訂を行う場合は、あらかじめ医学部医倫理委員会の承認を得る。

12.　研究の費用

12.1.　 研究資金及び利益相反

　本研究は、研究責任者が所属する大学の運営費交付金、および一般社団法人日本作業療法士協会研究助成金（予定）を用いて実施する。

12.2.　被験者の費用負担

　本研究で用いる治療および実施する検査は保険診療内で行われるため、研究に参加することによる患者の費用負担は発生しない。

12.3.　健康被害への対応と補償

　本研究は、既に精神科病院や精神病床を有する一般病院等で実施されている精神科OTの効果を検証することを目的としている。その中で予測される有害事象は、OTが過度な賦活刺激として作用した場合、不安感や疲労感などが増すことである。必要な場合には被験者の保険診療内で検査や治療等、必要な処置を行う。

13.　研究期間と研究の終了・早期中止

13.1.　研究期間

症例登録期間：2015年11月2日～2017年3月31日

研究実施期間：2015年11月2日～2019年3月31日

13.2.　研究の終了

　最終登録被験者のデータ固定が終了した時点で本研究の終了とし、研究責任者は速やかに研究終了報告書を医学部長に提出する。

13.3.　研究の早期中止

　研究担当者は、以下の事項に該当する場合は、研究実施継続の可否を検討する。

1）被験者の組み入れが困難で、予定症例数に達することが極めて困難であると判断されたとき

2）予定症例数または予定期間に達する前に、研究の目的が達成されたとき

3）医学部医倫理委員会により、実施計画等の変更の指示があり、これを受入れることが困難と判断されたとき

　研究責任者は、医学部医倫理委員会により中止の勧告あるいは指示があった場合は、研究を中止する。また、研究の中止を決定した時は、速やかに医学部長にその理由とともに文書で報告する。

14.　医療機器等の保存及び使用方法並びに保存期間

　研究実施に係る試料等を取扱う際は、被験者の個人情報とは無関係の番号を付して管理し、被験者の秘密保護に十分配慮する。試料等を研究事務局等の関連機関に送付する場合はこの番号を使用し、被験者の個人情報が院外に漏れないよう十分配慮する。また、研究の結果を公表する際は、被験者を特定できる情報を含まないようにする。研究の目的以外に、研究で得られた被験者の試料等を使用しない。

15.　記録の保存

　研究責任者は、研究等の実施に係わる重要な文書（申請書類の控え、病院長からの通知文書、各種申請書・報告書の控、同意書、その他データの信頼性を保証するのに必要な書類または記録等）を、研究の中止または終了後5年が経過した日までの間保存し、その後は個人情報に注意してシュレッターにかけて廃棄する。作成したデータファイルは研究責任者がパスワードを設定した専用デバイスに保存し厳重に管理する。

16.　研究の公表と成果の帰属

16.1.　臨床試験登録

　本研究は、UMIN 臨床試験登録システム（<http://www.umin.ac.jp/ctr/index-j.htm）の>データベースへ臨床試験登録を行う。

16.2.　成果の帰属

　本研究の成果は、信州大学に帰属するものとする。研究責任者及び研究担当者は、本研究の成果を関連学会、論文等を通じて公表する。

17.　研究実施体制

　本研究は、以下の体制で実施する。

【研究責任者】

小林　正義　　信州大学大学院医学系研究科保健学専攻 教授

　　　　　　　〒390-8621　松本市旭3-1-1

　　　　　　　TEL/FAX：0263-37-2403、E-mail：mkobaya@shinshu-u.ac.jp

【研究事務局】

島田　　岳　　信州大学大学院医学系研究科保健学専攻博士後期課程 大学院生

　　　　　　　医療法人清泰会メンタルサポートそよかぜ病院 作業療法士

〒386-0401　長野県上田市塩川3057-1

　　　　　　　TEL：0268-35-0305、FAX：0268-35-0534、E-mail：ot@seitaikai.co.jp

【研究担当者】

島田　　岳　　信州大学大学院医学系研究科保健学専攻博士後期課程 大学院生

　　　　　　 医療法人清泰会メンタルサポートそよかぜ病院 作業療法士

〒386-0401　長野県上田市塩川3057-1

　　　　　　　TEL：0268-35-0305、FAX：0268-35-0534、E-mail：ot@seitaikai.co.jp

埴原　秋児　　信州大学大学院医学系研究科保健学専攻 教授

　　　　　　　〒390-8621　松本市旭3-1-1

　　　　　　　TEL/FAX：0263-37-3599

【研究協力者】

　研究協力者は共同研究施設において本研究計画による介入を行い、主要評価項目及び副次評価項目に関する試料を収集し、研究担当者に提出する。

吉田　朋孝　　医療法人清泰会メンタルサポートそよかぜ病院 理事長・院長

〒386-0401　長野県上田市塩川3057-1

　　　　　　　TEL：0268-35-0305、FAX：0268-35-0534

樋掛　忠彦　　長野県立こころの医療センター駒ヶ根 院長

荒井留美子　　長野県立こころの医療センター駒ヶ根 作業療法士

〒399-4101　長野県駒ケ根市下平2901

　　　　　　　TEL：0265-83-3181、FAX：0268-83-4158

松岡　孝一　　医療法人愛生会松岡病院 院長

石原　郁代　　医療法人愛生会松岡病院 作業療法士

　　　　　　　〒399-0011　長野県松本市寿北2-6-2

　　　　　　　TEL：0263-25-1093，FAX：0263-28-2338

武藤　　隆　　医療法人蜻蛉会南信病院 院長

杉村　直哉　　医療法人蜻蛉会南信病院 作業療法士

　　　　　　　〒399-4511　長野県上伊那郡南箕輪村8811

　　　　　　　TEL：0265-78-4161、FAX：0265-76-6923

村田　志保　　長野県厚生農業協同組合連合会 北アルプス医療センター あづみ病院 こころのホスピタル事業部長

大堀　愛美　　長野県厚生農業協同組合連合会 北アルプス医療センター あづみ病院 作業療法士

　　　　　　　〒399-8695　長野県北安曇郡池田町大字池田3207-1

　　　　　　　TEL：0261-62-3166、FAX：0261-62-2711

南風原　泰　　社会医療法人栗山会飯田病院 精神科部長

下岡　佑子　　社会医療法人栗山会飯田病院 作業療法士

　　　　　　　〒395-8505　長野県飯田市大通1-15

　　　　　　　TEL：0265-22-5150、FAX：0265-22-3988

【個人情報管理者】

西　　麻郁　　医療法人清泰会メンタルサポートそよかぜ病院 作業療法士

〒386-0401　長野県上田市塩川3057-1

　　　　　　　TEL：0268-35-0305、FAX：0268-35-0534

植木　麻希　　医療法人愛生会松岡病院 作業療法士

　　　　　　　〒399-0011　長野県松本市寿北2-6-2

　　　　　　　TEL：0263-25-1093，FAX：0263-28-2338

杉村　めぐみ　長野県立こころの医療センター駒ヶ根 作業療法士

〒399-4101　長野県駒ケ根市下平2901

　　　　　　　TEL：0265-83-3181、FAX：0268-83-4158

寺島亜友美　　医療法人蜻蛉会南信病院 作業療法士

　　　　　　　〒399-4511　長野県上伊那郡南箕輪村8811

　　　　　　　TEL：0265-78-4161、FAX：0265-76-6923

千葉　　大　　長野県厚生農業協同組合連合会 北アルプス医療センター あづみ病院 作業療法士

　　　　　　　〒399-8695　長野県北安曇郡池田町大字池田3207-1

　　　　　　　TEL：0261-62-3166、FAX：0261-62-2711

加藤　未来　　社会医療法人栗山会飯田病院 作業療法士

　　　　　　　〒395-8505　長野県飯田市大通1-15

　　　　　　　TEL：0265-22-5150、FAX：0265-22-3988

18.　参考資料・参考文献

1) World Health Organization. Mental Health Atlas 2011. Geneva: World Health Organization, 2011

2) OECD Publishing. OECD Health Data 2014. Paris: Organisation for Economic Co-operation and Development, 2014

3) Ministry of Health, Labor and Welfare. Patient Survey 2011. Tokyo; Ministry of Health, Labor and Welfare, 2012

4) Ministry of Health, Labor and Welfare. Survey of Medical Institutions 2013. Tokyo; Ministry of Health, Labor and Welfare, 2014

5) Gold JM, Harvey PD. Cognitive deficits in schizophrenia. Psychiatr Clin North Am 1993; 16: 295-312

6) Green MF. What are the functional consequences of neurocognitive deficits in schizophrenia ?. Am J Psychiatry 1996; 153: 321-30

7) Green MF, Kern RS, Braff DL, Mintz J. Neurocognitive deficits and functional outcome in schizophrenia: are we measuring the "right stuff" ? Schizophr Bull 2000; 26: 119-36

8) Green MF, Nuechterlein KH. Should schizophrenia be treated as a neurocognitive disorder?. Schizophr Bull 1999; 25: 309-19

9) Green MF, Kern RS, Heaton RK. Longitudinal studies of cognition and functional outcome in schizophrenia: implications for MATRICS. Schizophr Res 2004; 72: 41-51

10) Shimada T, Kobayashi M, Tomioka N. Effects of individual occupational therapy on cognitive impairment in schizophrenia. JOTR 2014; 33: 67-74 (in Japanese)

11) Shimada T, Kobayashi M, Nishi A, Ishiwata M, Yoshida T. Predictors of rehospitalization in schizophrenia: a retrospective cohort study. JOTR 2015; 34: 51-60 (in Japanese)

12) American Psychiatric Association. Diagnostic and Statistical Manual of Mental Disorders, 4th edition, text revision (DSM-IV-TR). Washington, DC, American Psychiatric Association, 2000

13) Fukushima S, Kobayashi M, Murata S, Tomioka N. Assessment of the early recovery stage for people with mental disabilities: reliability and validity of the inventory scale of daily activities for sub-acute in-patients. JOTR 2011; 30: 9-19 (in Japanese)

14) Kobayashi M, Shimada T, Fukushima S, Tomioka N. Assessment of subjective experience for patients with schizophrenia: selection of sub-items and factor structure of the inventory scale for mood and sense of fatigue (SMSF). JOTR 2011; 30: 698-706 (in Japanese)

15) Shimada T, Kobayashi M, Fukushima S, Tomioka N. Reliability and validity of the inventory scale for mood and sense of fatigue (SMSF). JOTR 2012; 31: 540-9 (in Japanese)

16) 日本作業療法士協会. 作業療法マニュアル41 精神障害の急性期作業療法と退院促進プログラム. 東京, 日本作業療法士協会, 2011

17) Keef RS, Goldberg TE, Harvey PD, Gold JM, Poe MP, Coughenour L. The brief assessment of cognition in schizophrenia: reliability, sensitivity, and comparison with a standard neurocognitive battery. Schizophr Res 2004; 68: 283-97

18) Kaneda Y, Sumiyoshi T, Keefe RS, Ishimoto Y, Numata S, Ohmori T. Brief assessment of cognition in schizophrenia: validation of the Japanese version. Psychiatry Clin Neurosci 2007; 61: 602-9

19) Keefe RS, Poe M, Walker TM, Kang JW, Harvey PD. The Schizophrenia Cognition Rating Scale: an interview-based assessment and its relationship to cognition, real-world functioning, and functional capacity. Am J Psychiatry 2006; 163: 426-32

20) Kaneda Y, Ueoka Y, Sumiyoshi T, Furukori N, Ito T, Higuchi Y, et al. The Schizophrenia Cognition Rating Scale Japanese Version (SCoRS-J). Seishin Igaku (Clin Psychiatry) 2010; 52: 1027-30 (in Japanese)

21) Birchwood M, Smith J, Cochrane R, Wetton S, Copestake S. The Social Functioning Scale. The development and validation of a new scale of social adjustment for use in family intervention programmes with schizophrenic patients. Br J Psychiatry 1990; 157: 853-9

22) Nemoto T, Fujii C, Miura U, Chino B, Kobayashi H, Yamazawa R, et al. Reliability and validity of the Social Functioning Scale Japanese version (SFS-J). JPN Bull Soc Psychiat 2008; 17: 188-95 (in Japanese)

23) Choi J, Mogami T, Medalia A. Intrinsic motivation inventory: an adapted measure for schizophrenia research. Schizophr Bull 2010; 36: 966-76

24) Kay SR, Fiszbein A, Opler LA. The positive and negative syndrome scale (PANSS) for schizophrenia. Schizophr Bull 1987; 13: 261-76

25) Jones SH, Thornicroft G, Coffey M, Dunn G: A brief mental health outcome scale-reliability and validity of the Global Assessment of Functioning (GAF). Br J Psychiatry 1995; 166: 654-9

26) [Attkisson CC](http://www.ncbi.nlm.nih.gov/pubmed/?term=Attkisson%20CC%5BAuthor%5D&cauthor=true&cauthor_uid=10259963), [Zwick R](http://www.ncbi.nlm.nih.gov/pubmed/?term=Zwick%20R%5BAuthor%5D&cauthor=true&cauthor_uid=10259963). The client satisfaction questionnaire. Psychometric properties and correlations with service utilization and psychotherapy outcome. [Eval Program Plann](http://www.ncbi.nlm.nih.gov/pubmed/10259963) 1982; 5: 233-7

27) Tachimori H, Ito H. Reliability and validity of the Japanese version of client satisfaction questionnaire. Seishin Igaku (Clin Psychiatry) 1999; 41: 711-7 (in Japanese)
