# Supplementary material for: A multicenter, randomized controlled trial of individualized occupational therapy for patients with schizophrenia in Japan
Source: PLoS One. 2018 Apr 5;13(4):e0193869. doi: 10.1371/journal.pone.0193869 (PMC5886394; doi:10.1371/journal.pone.0193869)
Supplement: S3 File — (DOCX) [file pone.0193869.s003.docx]

**Clinical Trial**

**A multicenter, randomized controlled trial of**

**individualized occupational therapy for**

**patients with schizophrenia in Japan**

**Trial Protocol**

Research director

Masayoshi Kobayashi, OTR, PhD

Sinshu University

1st edition (September 1, 2015)

Contents

0. Overview

0.1. Schema

0.2. Objective

0.3. Participant

0.4. Target sample size and trial period

0.5. Trial design

0.6. Assessment

0.7. Contact address

1. Objective

2. Background

3. Eligibility criteria

3.1. Inclusion criteria

3.2. Exclusion criteria

4. Methods

4.1. Trial design

4.2. Trial outline

4.3. Intervention

4.4. Recruitment and allocation method

4.5. Correspondence after study completion

5. Assessment outcomes

5.1. Primary outcomes

5.2. Key secondary outcomes

6. Adverse events

6.1. Definition of adverse events

6.1.1. Definition of adverse events

6.1.2. Definition of serious adverse events

6.2. Correspondence to participants at adverse event manifestation

6.3. Evaluation and reporting of adverse events

6.4. Predicted adverse events

7. Target sample size

8. Statistical methods

8.1. Setting evidence of target sample size

8.2. Statistical methods

8.3. Analysis items and methods

8.3.1. Outline of analysis target

8.3.2. Hypothesis verification analysis

8.3.3. Hypothesized exploratory analysis

9. Monitoring

10. Ethics

10.1. Adherence rules

10.2. Informed consent

10.3. Protection of personal information

11. Change of trial plan

12. Funding

12.1. Funding and conflicts of interest

12.2. Finance burden on participants

12.3. Correspondence to health damage and compensation

13. Trial period and trial termination

13.1. Trial period

13.2. Trial termination

13.3. Early termination of trial

14. Storage and use of medical devices and storage period

15. Preservation of records

16. Trial publication and results attribution

16.1. Clinical trial registration

16.2. Results attribution

17. Trial organization

18. References

0. Overview

0.1. Schema

Recently hospitalized patients with schizophrenia or schizoaffective disorder

Registration and randomized allocation

Target sample size: 150 (Intervention group, 75; Control group, 75)

Registration period: November 2, 2015–March 31, 2017

Allocation adjustment factor: age, sex, number of hospitalizations

Intervention group

Individualized occupational therapy

+

Group occupational therapy

Control group

Group occupational therapy alone

Post assessments: Discharge or 3 months following hospitalization

Intervention group

Control group

2-year follow-up after discharge

0.2. Objective

(1) To examine the feasibility and effectiveness of individual occupational therapy for cognitive functioning and other outcomes in patients with schizophrenia

(2) To explore the factors influencing rehospitalization of patients with schizophrenia

0.3. Participant

Recently hospitalized patients with schizophrenia or schizoaffective disorder

0.4. Target sample size and trial period

Target sample size: 150 (Intervention group, 75; Control group, 75)

Registration period: November 2, 2015–March 31, 2017

Trial period: November 2, 2015–March 31, 2019

0.5. Trial design

- Design: a multicenter, open-labeled, blinded-endpoint, randomized controlled trial
- Types of controls: treatment as usual (group occupational therapy)
- Randomization: randomized by minimization method after stratification by sex, age, number of hospitalizations
- Blinding: treatment assignment not known in advance by study personnel; assessments conducted by trained evaluators blind to treatment assignment

0.6. Assessment

- Primary outcomes: cognitive functioning
- Key secondary outcomes: intrinsic motivation, social functioning, medication adherence, symptoms, treatment satisfaction, and rehospitalization (rate)

0.7. Contact address

[Research director]

Masayoshi Kobayashi, OTR, PhD

Graduate School of Medicine, Shinshu University, 3-1-1, Asahi, Matsumoto, Nagano, Japan

Tel: +81-268-37-2403 Fax: +81-268-37-2403

E-mail: mkobaya@shinshu-u.ac.jp

[Research office]

Takeshi Shimada, OTR, MHS

Medical Corporation Seitaikai Mental Support Soyokaze Hospital, 3057-1, Shiogawa, Ueda, Nagano, Japan

Graduate School of Medicine, Shinshu University, 3-1-1, Asahi, Matsumoto, Nagano, Japan

TEL: +81-268-35-0305 FAX: +81-268-35-0534

E-mail: ot@seitaikai.co.jp

1. Objective

(1) To examine the feasibility and effectiveness of individual occupational therapy for cognitive functioning and other outcomes in patients with schizophrenia

(2) To explore the factors influencing rehospitalization of patients with schizophrenia

2. Background

Psychosocial treatments designed to remediate or bypass cognitive impairments are important to pursue to maximize outcomes for patients with schizophrenia [1–6]. Cognitive impairment is a core feature of schizophrenia [7–10], and previous studies have demonstrated that it is strongly correlated with daily functioning and other functional outcomes [7–12]. Because of the importance of cognitive impairment in schizophrenia, it has been proposed as an appropriate target for intervention [4, 5, 11]. Antipsychotic treatment reduces symptoms of schizophrenia; however, it does little to improve cognitive impairment [5, 13, 14]. Therefore, it is important to improve cognitive impairment in patients with schizophrenia using psychosocial treatment [5].

In Japan, occupational therapy (OT) is a required component of psychosocial treatment that helps therapists provide short-term inpatient care for patients with acute schizophrenia. Furthermore, it is believed that individualized OT (IOT) is needed to achieve this goal. However, the existing medical fee system for psychiatric OT in Japan was developed based on the assumption that group treatment was the standard for long-term hospitalized patients in 1974. This system is very different from current psychiatric treatment and rehabilitation, which are based on personalized support for short-term hospitalized patients. For this reason, an activity-oriented group OT (GOT) intervention is widely practiced for patients with schizophrenia in several Japanese psychiatric hospitals, and individualized OT is not often implemented in Japan [15].

Several studies have examined the effects of OT on patients with schizophrenia. Many of these reported that OT can improve symptoms, and combined interventions of OT and pharmacological treatment can improve symptoms more effectively than pharmacological treatment alone [16, 17]. However, the effectiveness of OT for cognitive impairment in patients with schizophrenia has not been sufficiently verified.

The IOT program is an intervention that we developed as part of an OT program based on individualized interventions for inpatients with acute schizophrenia, which facilitates proactive participation in treatment and improving outcomes [18]. IOT strategies are tailored to enhance cognitive functioning and prompt adaptive behaviors to maximize the functional outcomes of patients with schizophrenia. It consists of a combination of effective psychosocial treatment programs that are very relevant to OT practice, including motivational interviewing, self-monitoring, individualized visits, handicraft activities, individualized psychoeducation, and discharge planning. It was implemented on a one-on-one basis with occupational therapists. In the program, we used constitutive handicraft activities with the individualized coaching of occupational therapists as a means of improving the cognitive impairment associated with schizophrenia. Handicraft activities have been widely used as OT therapeutic modalities [19–21].

The finding of our previous pilot study concerning the IOT program provided preliminary support for the feasibility of IOT for helping patients with schizophrenia who were enrolled in an OT program at a Japanese psychiatric hospital and its beneficial effects on improving cognitive impairment and symptoms for patients with schizophrenia [18] and decreasing rehospitalization rate after discharge with one-year follow-up [22]. However, it had several limitations, including being conducted in only one site and having selection biases because the study design was a non-randomized controlled trial in which participants were assigned to either the IOT + GOT or GOT alone group by voluntary selection according to their preferences. In addition, the outcomes used in the study were not sufficient to examine the effects of IOT because only cognitive functioning, social functioning, and symptoms were measured.

Therefore, the present study reexamined the feasibility and effectiveness of IOT for cognitive functioning and other outcomes including social functioning, intrinsic motivation, medication adherence, symptoms, and treatment satisfaction in patients with schizophrenia using a multicenter, randomized, controlled trial design. Moreover, a follow-up study is planned to explore the factors influencing rehospitalization of patients with schizophrenia including the OT interventions and to examine whether IOT is useful to prevent rehospitalization of patients with schizophrenia in Japan.

3. Eligibility criteria

3.1. Inclusion criteria

- aged 20–65 years
- recently hospitalized patients in the psychiatric hospital
- a diagnosis of schizophrenia or schizoaffective disorder based on the DSM-5 criteria [23]

3.2. Exclusion criteria

- a diagnosis of mental retardation or alcohol or drug disorders (abuse or dependence)
- any current or history of neurological disorders including head injury, cerebral vascular disorders, epilepsy, or dementia
- the need for a specific individual intervention for a physical dysfunction

4. Methods

4.1. Trial design

- Design: a multicenter, open-labeled, blinded-endpoint, randomized controlled trial
- Types of controls: treatment as usual (GOT)
- Randomization: Randomized by minimization method after stratification by sex (male/female), age (20–29, 30–39, 40–49, 50–59, and 60–65 years), and number of hospitalizations (≤3 or ≥4)
- Blinding: treatment assignment not known in advance by study personnel; assessments conducted by trained evaluators blind to treatment assignment

4.2. Trial outline

This study evaluates the impact of adding IOT to a GOT program as usual for outcomes in recently hospitalized patients with schizophrenia in Japanese psychiatric hospitals compared with GOT alone. Participants are randomly assigned to the GOT + IOT or GOT alone group. Outcomes are administered at baseline and discharge or 3 months following hospitalization including the Brief Assessment of Cognition in Schizophrenia Japanese version (BACS-J), Schizophrenia Cognition Rating Scale Japanese version (SCoRS-J), Social Functioning Scale Japanese version (SFS-J), Global Assessment of Functioning scale (GAF), Intrinsic Motivation Inventory Japanese version (IMI-J), Morisky Medication Adherence Scale-8 (MMAS-8), Positive and Negative Syndrome Scale (PANSS), and Japanese version of the Client Satisfaction Questionnaire-8 (CSQ-8J). Moreover, a follow-up study is planned to explore the factors influencing rehospitalization, including the type of OT implemented during hospitalization.

4.3. Intervention

Time and frequency of OT implementation for both the GOT + IOT and GOT only groups were adjusted according to recovery progress; however, these were generally 1–2 hours at a time, 3–5 times per week. Notably, more than half of the OT time was devoted to IOT in the GOT + IOT group. Occupational therapists provided support, such as consultations concerning living challenges, preparation support for discharge, and provision of information concerning available social resources and community services for all participants, regardless of OT program type (GOT + IOT versus GOT alone). In addition, support personnel, including hospital staff other than occupational therapists, provided necessary support for all participants regardless of OT program type.

4. 3. 1. IOT program

The IOT program is a psychosocial program that we developed to facilitate proactive participation in treatment and improve cognitive functioning and other outcomes for inpatients with acute schizophrenia. It consists of a combination of effective psychosocial treatment programs that are very relevant to OT practice: motivational interviewing, self-monitoring, individualized visits, handicraft activities, individualized psychoeducation, and discharge planning. The IOT is summarized in Table 1. It is provided on a one-on-one basis with occupational therapists in charge and is tailored to each participant. Therapeutic structure factors such as time, frequency, and place are set for everyone.

Motivational interviewing was regularly implemented 2–3 times per week in 15–30-minute sessions to improve motivational deficits and enhance motivation for treatment. Occupational therapists confirm the course of OT and its effects and modify OT planning and goals. Furthermore, occupational therapists promote independence through OT by addressing individuals’ hospitalization and discharge challenges.

A self-monitoring program was implemented on a weekly basis to improve self–body disturbances, subjective experience deficits, and metacognition. Recovery from self–body disturbance was promoted through physical exercise, such as stretching, on a one-on-one basis with an occupational therapist. Positive feedback was provided to improve confidence, self-efficacy, and subjective experience deficits by addressing challenges and completing specific occupational activities. Subjective experience and metacognition in schizophrenia are moderators of cognitive and social functioning and affect insight.

Individualized visits were used as a support strategy for conducting activities of daily living away from the hospital. During the first half of their hospitalization, this was implemented 2–3 times per week. During the second half of their hospitalization, support was provided as necessary in a community setting for going out, social resource utilization, and home visits prior to discharge.

The therapeutic use of handicraft activities is one feature of OT for schizophrenia. Constructive handicraft activities with clear procedures and good feasibility, such as Japanese paper collages, plastic models, Japanese paper crafts, and jigsaw puzzles, were used in the handicraft activities program. Handicraft activities were implemented 3–5 times per week. Implementation time was about 30 minutes per session at the start of OT and was gradually extended to about 60 minutes. To activate cognitive functioning such as vigilance, attention, executive function, and matching function, the patients were asked to attend to, concentrate on, precisely perform, and efficiently use the craft tools along with the individualized coaching. Occupational therapists observed the occupational performance characteristics of each patient. Interventions bridging the gap between improvements in cognitive impairment, as observed in OT situations and daily functioning, were implemented.

Illness management and relapse prevention programs were implemented 1–2 times per week for 45–60 minutes each, and a crisis plan was developed as part of the individualized psychoeducation program. Occupational therapists supported each participant in identifying relapse signs and finding practical coping methods to address them. A crisis plan was developed and shared with family members and other support persons through care conferences.

Post-discharge care and weekly action plans, which included information on how to manage living time within a community setting, were developed to promote a smooth transition from the hospital setting to community living. These developments were conducted in collaboration with each patient 1–2 times per week during the second half of the hospitalization. Skills training included training focused on living skills such as how to manage living time, money, and diet preparation. Interpersonal skills training focused on things such as how to greet people and ask for help if the need arose. These were both implemented to improve aspects of each participant’s daily functioning, such as instrumental daily living activities, education, and work with recovery states, and each required 1–2 times per week during the second half of hospitalization.

4. 3. 2. GOT program

The GOT program is an activity-oriented group treatment program usually implemented based on a weekly OT schedule at each study site. The GOT includes the following programs: a group physical fitness program (stretching exercise, relaxation, and breathing), a group handicraft activities program in which participants choose and participate in desired activities programs; a group cooking program, a group music program (music appreciation and singing), a recreation program, and a group psychoeducation program. In the group handicraft activities program, each participant voluntarily completed the craft activities based on their preferences. The participants voluntarily selected any desired program among these and participated at an individualized rate. These programs were held either in hospital ward halls or OT rooms. Several patients simultaneously participated in each program and addressed activities in accordance with the program.

Table 1. IOT program summary

| Program | Description |
| --- | --- |
| Motivational interviewing | - Regular implementation of motivational interviewing 2–3 times per week in 15–30 minute sessions - Intervention for improving motivational deficits - Promoting independence for OT by addressing the individual’s challenges while in hospital and after discharge |
| Self-monitoring | - Implementation on a weekly basis - Physical exercise on a one-on-one basis with an occupational therapist - Positive feedback for improving subjective experience deficits - Metacognitive training |
| Individualized visits | - Support strategy for carrying out activities of daily living away from the hospital room 2–3 times per week during the first half of the hospitalization - Support provided, as necessary, for going out, utilizing social resources, and home visits prior to discharge in a community setting during the second half of the hospitalization |
| Handicraft activities | - Frequency 3–5 times per week - **Implementation time about 30 minutes per session at the start of OT, gradually extended to about 60 minutes** - **Utilization of** constructive activities - Providing guidance on asking participants to attend to, concentrate on, precisely perform, and efficiently use instruments and materials - Bridging improvements in cognitive impairment and daily functioning |
| Individualized psychoeducation | - Illness management program and relapse prevention program implemented 1–2 times per week for 45–60 minutes - Development of a crisis plan |
| Discharge planning | - Development of a post-discharge care plan and weekly action plan 1–2 times per week during the second half of the hospitalization - Skills training 1–2 times per week during the second half of the hospitalization |
| IOT: individualized occupational therapy; OT: occupational therapy. | |

4.4. Recruitment and Allocation method

Participants are recruited through referrals from occupational therapists at each study center. Following completion of the baseline assessment, participants are randomized to either the GOT + IOT or the GOT alone groups by the project coordinator using a computer-generated randomization program. Randomization is stratified by sex (male/female), age (20–29, 30–39, 40–49, 50–59, and 60–65 years), and number of hospital stays (≤3 or ≥4). Within each stratum, patients are randomly assigned 1:1 to the GOT + IOT or the GOT alone groups.

4.5. Correspondence after study completion

The research director will provide medical treatment considered most appropriate for the participant after study completion.

5. Assessment outcomes

- BACS-J [24, 25]
- SCoRS-J [26-28]
- IMI-J [29]
- SFS-J [30, 31]
- GAF [32]
- MMAS-8 [33, 34]
- PANSS [35]
- CSQ-8J [36, 37]
- Rehospitalization rate

6. Adverse events

6.1. Definition of adverse events

6.1.1. Definition of adverse events

An adverse event is any undesirable medical event that occurred in participants who received OT, with or without a causal relationship with OT.

6.1.2. Definition of serious adverse events

A serious adverse event is an adverse event that falls under the following categories:

1. Death

2. Danger of leading to death

3. Hospitalization for treatment or extension of hospitalization required

4. Handicap

5. Danger of leading to handicap

6. Other, severe in accordance with 1 to 5

7. Causes congenital disease or abnormality in later generations

6.2. Correspondence to participants at adverse event manifestation

This study examines the effect of the IOT program that is already implemented at the psychiatric hospitals in Japan. Therefore, there is little possibility of adverse events occurring in this study. However, when an adverse event occurs, the research director or research staff immediately takes appropriate treatments and report them in the medical record and case report. Additionally, in case of stopping IOT or when treatment for adverse event becomes necessary, we inform the participant to that effect.

6.3. Evaluation and reporting of adverse events

Research staff promptly inform the medical department chief of all serious adverse events that occur during the study period and serious adverse events suspected of being associated with OT that developed within 2 days of study termination (discontinuation). In accordance with serious adverse events, we promptly report them as a first report (emergency report), and a second report (detailed report) shall be made within 7 days. If necessary, we also report in the third and subsequent reports. For other adverse events, the research staff properly include them in the medical record and case report.

6.4. Predicted adverse events

It is possible to temporarily increase feelings of anxiety and fatigue in the acute phase of schizophrenia, because IOT acts as an excessive stimulus.

7. Target sample size

The planned sample size was 150 patients with 75 randomized patients per treatment group.

8. Statistical methods

8.1. Setting evidence of target sample size

The planned sample size is 150 patients with 75 randomized patients per treatment group, each patient contributing at least 1 baseline and 1 post-randomization assessment per treatment arm. This calculation is based on a two-sided test with σ = 2.286, σ x = 0.490, λ = 1.154, type I error rate of 5, and 80% power to show statistically significant differences between treatments based on results of Shimada’s pilot follow-up study of IOT [22]. The result of the calculation showed the needed sample size to be 130 patients with 65 patients per treatment group. However, an assumed patient dropout rate of approximately 20% led to a randomization target of 75 patients per treatment group.

8.2. Statistical methods

Analyses of patients’ outcomes are conducted on an intention-to-treat basis.

8.3. Analysis items and methods

The outline of statistical analysis is shown below. A test result was considered significant if p < .05 for a two-sided test.

8.3.1. Outline of analysis target

(1) Component of analysis target group

The numbers of registered cases, eligible cases, treatment initiation cases, and analyzed cases are calculated for each treatment group.

(2) Demographics and baseline data

For demographics and baseline data, summary statistics are calculated for each treatment group.

(3) Treatment information

For the method of treatment (OT), the appropriate intervention method is described by treatment group.

8.3.2. Hypothesis verification analysis

First, we compare the groups at baseline on demographics, cognition, and other measures using t-tests for the continuous variables and χ^2^ analyses for categorical variables. Second, the feasibility and safety assessments are summarized using descriptive statistics. We compute the percentage of participants exposed to the IOT program, the average number of OT sessions, and the length of OT intervention. Third, to examine group differences in cognitive functioning outcomes, we include the BACS-J and SCoRS-J over time (baseline and post assessment time) by treatment group (GOT + IOT and GOT only) using the linear mixed effects models with repeated measures with the participants as the random effect; the treatment, assessment time points, and interaction of treatment by time as fixed effects; and the baseline scores and the baseline IMI-J total score as covariates in the model. Moreover, we examine group differences in intrinsic motivation (IMI-J), social functioning (GAF), medication adherence (MMAS-8), and symptomatology (PANSS) over time (baseline and post assessment time) by treatment group (GOT + IOT and GOT only) using the linear mixed effects models with repeated measures with the participants as the random effect; the treatment, assessment time points, and interaction of treatment by time as fixed effects; and the baseline scores as covariates in the model. For significant effects, we report effect size as Cohen’s d.

8.3.3. Hypothesized exploratory analysis

First, we compare groups on the demographics, each assessment score on hospitalization, the community living conditions after discharge, and the contents of outpatient treatment using t-tests for continuous variables and χ^2^ analyses for categorical variables. Variables that satisfied p < .05 were selected for further analysis using logistic regression analysis to control confounding factors. Second, we calculate Pearson's correlation coefficient between each variable that had significant differences in previous univariate analyses to exclude the influence of multicollinearity. Third, we conduct a binomial logistic regression analysis to determine the factors influencing rehospitalization and to calculate the odds ratio (OR) and 95% confidence interval (95% CI) after controlling simultaneously for potential confounders with rehospitalization as the dependent variable and the variables selected in previous analyses as the independent variable.

9. Monitoring

The monitoring staff prepares a monitoring plan sheet, summarizes the problems periodically (monthly) according to the plan by type, and submit to the research director. The research director confirms the report and its details and issues a warning document and revises the study implementation plan if a problem recurs.

[Monitoring items]

(1) Consistency with eligibility criteria

(2) Consistency with treatment plan

(3) Unreported serious adverse events

(4) Consistency between generated adverse events and discontinuance criteria

(5) Essential inspection and not implemented observation items

(6) Other problems such as protocol deviation

10. Ethics

10.1. Adherence rules

Study staff comply with "World Medical Association Declaration of Helsinki" and "Ethical Guidelines for Medicine Research Involving Human Subjects."

10.2. Informed consent

The research staff and collaborators hand out the consent explanation document, which obtains approval by the ethics committee for the participant; sufficiently explain in writing and verbally, and acquire the consent of the trial participant’s free and intentional participation.

The research staff and collaborators promptly provide information to the participant when information that affects the consent of the participant is obtained or when an implementation plan that affects the consent is changed. Then, the research staff and collaborators confirm the intention of the participant whether to participate in the research in advance. In addition, the research staff and collaborators revise the agreement explanation document with the approval of the ethics committee in advance and obtain the agreement of the participant again.

The consent explanation document includes the following contents:

(1) That participation is voluntary, that one does not suffer disadvantages for failing to agree, that consent can be withdrawn

(2) Background, objective, participant, methods, study period, planned sample size

(3) Expected benefits of participating in the study and disadvantage that can occur

(4) Viewing handling of samples including personal information, retention period and disposal method, study method, etc.

(5) Presentation of study results and handling when patent occurs

(6) Expense burden of participants, source of study funding, and conflicts of interest

(7) Consultation window (contact address) such as trial organization, inquiries about research, complaints, etc.

(8) Response in case of health damage to the participant and presence or absence of compensation

10.3. Protection of personal information

When dealing with data related to the study, personal information managers not directly involved in this study are managed by attaching numbers unrelated to the participants’ personal information, and careful consideration is given to the confidentiality of the participants. When researchers analyze data, we use this number and give due consideration so that the participants’ personal information does not leak to the outside. In addition, protection of personal information from each site is carried out by the occupational therapist in charge, and only the data to which the personal information is deleted are sent to the research office. When publishing the results of the trial, no information that can identify participants is included. The data are used only in the study.

11. Change of trial plan

In case of changing or revising the research plan and consent explanation document of this study, we obtain approval from the ethics committee of Shinshu University and all sites’ ethics committees.

12. Funding

12.1. Funding and conflicts of interest

This study is supported by the Japanese Association of Occupational Therapists (2016-01). The funders had no role in study design, data collection and analysis, decision to publish, or preparation of the manuscript.

12.2. Finance burden on participants

There is no cost burden on the participants.

12.3. Correspondence to health damage and compensation

This study examines the effect of OT that has already been implemented in the Japanese psychiatric hospital. The predicted adverse event is increasing feelings of anxiety and fatigue. If necessary, the necessary treatment is provided within the health insurance of participants.

13. Trial period and trial termination

13.1. Trial period

Registration period: November 2, 2015–March 31, 2017

Trial period: November 2, 2015–March 31, 2019

13.2. Trial termination

This study is completed when data fixing of the last registered participant is completed, and the research director promptly submits a research termination report to the medical department chief.

13.3. Early termination of trial

If the study participant falls under the following circumstances, the research staff will consider whether to continue the study.

(1) When judging that it is difficult to incorporate participants and it is extremely difficult to reach the number of planned sample size

(2) When the objective of the study is achieved before reaching the planned sample size or study period

(3) When the ethics committee instructs the change of the study plan etc. and it is difficult to accept it

The research director will stop the trial if there is a recommendation or order to cancel by the ethics committee. Additionally, when it is decided to stop the trial, we will promptly report to the medical department chief with a written reason.

14. Storage and use of medical devices and storage period

When handling the data related to the study, we attach a number unrelated to the participant's personal information and manage it carefully to protect the participant's confidentiality. When sending data to relevant organizations of the research secretariat, we use this number and give due consideration so that the participant's personal information does not leak out of the research center. Additionally, when publishing the results of the study, we do not include personal information. We do not use data of participants obtained by research other than the study purpose.

15. Preservation of records

The research director preserves important documents in the 5 years since the cancellation or the study termination and then discards them by shredding any personal information. The created data file is saved in a dedicated, strictly managed device for which the research director has set a password.

16. Trial publication and results attribution

16.1. Clinical trial registration

This study is registered in the database of the UMIN clinical trial registration system (<http://www.umin.ac.jp/ctr/index-j.htm>).

16.2. Results attribution

The results of this study belong to Shinshu University. The research director and research staff will publish the results of this study through related academic societies, papers, and so on.

17. Trial organization

[Research contact person]

Masayoshi Kobayashi, OTR, PhD

Graduate School of Medicine, Shinshu University, 3-1-1, Asahi, Matsumoto, Nagano, Japan

E-mail: mkobaya@shinshu-u.ac.jp

Tel: +81-268-37-2403 Fax: +81-268-37-2403

E-mail：mkobaya@shinshu-u.ac.jp

[Research office]

Takeshi Shimada, OTR, MHS

E-mail: shimadatakeshi0703@yahoo.co.jp

Medical Corporation Seitaikai Mental Support Soyokaze Hospital, Nagano, Japan

Graduate School of Medicine, Shinshu University, Nagano, Japan

TEL: +81-268-35-0305 FAX: +81-268-35-0534

E-mail: ot@seitaikai.co.jp

[Research staff]

Takeshi Shimada, OTR, MHS

Medical Corporation Seitaikai Mental Support Soyokaze Hospital, Nagano, Japan

Graduate School of Medicine, Shinshu University, Nagano, Japan

Tokiji Hanihara, MD, PhD

Graduate School of Medicine, Shinshu University, 3-1-1, Asahi, Matsumoto, Nagano, Japan

[Research collaborators]

Tomotaka Yoshida, MD

Medical Corporation Seitaikai Mental Support Soyokaze Hospital, Nagano, Japan

Rumiko Arai, OTR

Nagano Prefectural Mental Wellness Center Komagane, Nagano, Japan

Ikuyo Ishihara, OTR

Medical Corporation Aiseikai Matsuoka Hospital, Nagano, Japan

Naoya Sugimura, OTR, MHS

Medical Corporation Akitsukai Nanshin Hospital, Nagano, Japan

Manami Ohori, OTR

North Alps Medical Center Azumi Hospital, Nagano, Japan

Yuko Shimooka, OTR

Social Medical Corporation Ritsuzankai Iida Hospital, Nagano, Japan

[Personal information manager]

Ai Nishi, OTR

Medical Corporation Seitaikai Mental Support Soyokaze Hospital, Nagano, Japan

Maki Ueki, OTR

Medical Corporation Aiseikai Matsuoka Hospital, Nagano, Japan

Megumi Sugimura, OTR

Nagano Prefectural Mental Wellness Center Komagane, Nagano, Japan

Ayumi Terashima, OTR

Medical Corporation Akitsukai Nanshin Hospital, Nagano, Japan

Dai Chiba, OTR

North Alps Medical Center Azumi Hospital, Nagano, Japan

Miku Kato, OTR

Social Medical Corporation Ritsuzankai Iida Hospital, Nagano, Japan

18. References

1. Velligan DI, Bow-Thomas CC, Huntzinger C, Ritch J, Ledbetter N, Prihoda TJ, Miller AL. Randomized controlled trial of the use of compensatory strategies to enhance adaptive functioning in outpatients with schizophrenia. Am J Psychiatry. 2000; 157: 1317–1323.

2. Kern RS, Green MF, Mitchell S, Kopelowicz A, Mintz J, Liberman RP. Extensions of errorless learning for social problem-solving deficits in schizophrenia. Am J Psychiatry. 2005; 162: 513–519.

3. Haddock G, Lewis S. Psychological interventions in early psychosis. Schizophr Bull. 2005; 31: 697–704.

4. Kurtz MM. Neurocognition as a predictor of response to evidence-based psychosocial interventions in schizophrenia: what is the state of the evidence? Clin Psychol Rev. 2011; 31: 663–672.

5. Keefe RS, Harvey PD. Cognitive impairment in schizophrenia. Handb Exp Pharmacol. 2012; 213: 11–37.

6. Nuechterlein KH, Ventura J, Subotnik KL, Hayata JN, Medalia A, Bell MD. Developing a cognitive training strategy for first-episode schizophrenia: integrating bottom-up and top-down approaches. Am J Psychiatr Rehabil. 2014; 17: 225–253

7. Green MF. What are the functional consequences of neurocognitive deficits in schizophrenia? Am J Psychiatry. 1996; 153: 321–330.

8. Green MF, Harvey PD. Cognition in schizophrenia: past, present, and future. Schizophr Res Cogn. 2014; 1: e1–e9.

9. Lepage M, Bodnar M, Bowie CR. Neurocognition: clinical and functional outcomes in schizophrenia. Can J Psychiatry. 2014; 59: 5–12.

10. Harvey PD, Green MF, Keefe RS, Velligan DI. Cognitive functioning in schizophrenia: a consensus statement on its role in the definition and evaluation of effective treatments for the illness. J Clin Psychiatry. 2004; 65: 361–372.

11. Szöke A, Trandafir A, Dupont ME, Méary A, Schürhoff F, Leboyer M. Longitudinal studies of cognition in schizophrenia: meta-analysis. Br J Psychiatry. 2008; 192: 248–257.

12. Kahn RS, Keefe RS. Schizophrenia is a cognitive illness: time for a change in focus. JAMA Psychiatry. 2013; 70: 1107–1112.

13. Woodward ND, Purdon SE, Meltzer HY, Zald DH. A meta-analysis of neuropsychological change to clozapine, olanzapine, quetiapine, and risperidone in schizophrenia. Int J Neuropsychopharmacol. 2005; 8: 457–472.

14. Keefe RS, Bilder RM, Davis SM, Harvey PD, Palmer BW, Gold JM, Meltzer HY, Green MF, Capuano G, Stroup TS, McEvoy JP, Swartz MS, Rosenheck RA, Perkins DO, Davis CE, Hsiao JK, Lieberman JA; CATIE Investigators; Neurocognitive Working Group. Neurocognitive effects of antipsychotic medications in patients with chronic schizophrenia in the CATIE Trial. Arch Gen Psychiatry. 2007; 64: 633–647.

15. Kayama A, Kobayashi M, Tsurumi T. Occupational therapy to support daily living for people with mental disorders-from hospital acute care to community based practice, 2nd ed. Tokyo: Ishiyaku Publishers; 2014. In Japanese

16. Foruzandeh N, Parvin N. Occupational therapy for inpatients with chronic schizophrenia: a pilot randomized controlled trial. Jpn J Nurs Sci. 2013; 10: 136–141.

17. Hoshii J, Yotsumoto K, Tatsumi E, Tanaka C, Mori T, Hashimoto T. Subject-chosen activities in occupational therapy for the improvement of psychiatric symptoms of inpatients with chronic schizophrenia: a controlled trial. Clin Rehabil. 2013; 27: 638–645.

18. Shimada T, Nishi A, Yoshida T, Tanaka S, Kobayashi M. Development of an individualized occupational therapy programme and its effects on the neurocognition, symptoms and social functioning of patients with schizophrenia. Occup Ther Int. 2016; 23: 425–435.

19. Fidler GS. From crafts to competence. Am J Occup Ther. 1981; 35: 567–573.

20. Allen CK. Occupational therapy for psychiatric diseases: measurement and management of cognitive disability. Boston: Little, Brown; 1985.

21. Horghagen S, Fostvedt B, Alsaker S. Craft activities in groups at meeting places: supporting mental health users’ everyday occupations. Scand J Occup Ther. 2014; 21: 145–152.

22. Shimada T, Nishi A, Yoshida T, Tanaka S, Kobayashi M. Factors influencing rehospitalisation of patients with schizophrenia in Japan: a 1-year longitudinal study. Hong Kong J Occup Th. 2016; 28: 7–14

23. American Psychiatric Association. Diagnostic and Statistical Manual of Mental Disorders, 5th edition (DSM-5). Washington DC: American Psychiatric Association; 2013.

24. Keefe RS, Goldberg TE, Harvey PD, Gold JM, Poe MP, Coughenour L. The Brief Assessment of Cognition in Schizophrenia: reliability, sensitivity, and comparison with a standard neurocognitive battery. Schizophr Res. 2004; 68: 283–297.

25. Kaneda Y, Sumiyoshi T, Keefe R, Ishimoto Y, Numata S, Ohmori T. Brief assessment of cognition in schizophrenia: validation of the Japanese version. Psychiatry Clin Neurosci. 2007; 61: 602–609.

26. Keefe RS, Poe M, Walker TM, Kang JW, Harvey PD. The Schizophrenia Cognition Rating Scale: an interview-based assessment and its relationship to cognition, real-world functioning, and functional capacity. Am J Psychiatry. 2006; 163: 426–432.

27. Kaneda Y, Ueoka Y, Sumiyoshi T, Furukori N, Ito T, Higuchi Y, et al. The Schizophrenia Cognition Rating Scale Japanese Version (SCoRS-J). Clin Psychiatry. 2010; 52: 1027–1030. In Japanese

28. Keefe RS, Davis VG, Spagnola NB, Hilt D, Dgetluck N, Ruse S, Patterson TD, Narasimhan M, Harvey PD. Reliability, validity and treatment sensitivity of the Schizophrenia Cognition Rating Scale. Eur Neuropsychopharmacol. 2015; 25: 176–184.

29. Choi J, Mogami T, Medalia A. Intrinsic Motivation Inventory: an adapted measure for schizophrenia research. Schizophr Bull. 2010; 36: 966–976.

30. Birchwood M, Smith J, Cochrane R, Wetton S, Copestake S. The Social Functioning Scale. The development and validation of a new scale of social adjustment for use in family intervention programmes with schizophrenic patients. Br J Psychiatry. 1990; 157: 853–859.

31. Nemoto T, Fujii C, Miura U, Chino B, Kobayashi H, Yamazawa R, Murakami M, Kashima H, Mizuno M. Reliability and validity of the Social Functioning Scale Japanese version (SFS-J). JPN Bull Soc Psychiat. 2008; 17: 188–195. In Japanese

32. American Psychiatric Association. Diagnostic and Statistical Manual of Mental Disorders, 4th edition, text revision (DSM-IV-TR). Washington DC: American Psychiatric Association; 2000.

33. Morisky DE, Green LW, Levine DM. Concurrent and predictive validity of a self-reported measure of medication adherence. Med Care. 1986; 24: 67–74.

34. Morisky DE, Ang A, Krousel-Wood M, Ward HJ. Predictive validity of a medication adherence measure in an outpatient setting. J Clin Hypertens. 2008; 10: 348–354.

35. Kay SR, Fiszbein A, Opler LA. The Positive and Negative Syndrome Scale (PANSS) for schizophrenia. Schizophr Bull. 1987; 13: 261–276.

36. Attkisson CC, Zwick R. The client satisfaction questionnaire. Psychometric properties and correlations with service utilization and psychotherapy outcome. Eval Program Plann. 1982; 5: 233–237.

37. Tachimori H, Ito H. Reliability and validity of the Japanese version of client satisfaction questionnaire. Clin Psychiatry. 1999; 41: 711–717. In Japanese
